# The Membrane-Associated Transcription Factor NAC089 Controls ER-Stress-Induced Programmed Cell Death in Plants

**DOI:** 10.1371/journal.pgen.1004243

**Published:** 2014-03-27

**Authors:** Zheng-Ting Yang, Mei-Jing Wang, Ling Sun, Sun-Jie Lu, Dong-Ling Bi, Le Sun, Ze-Ting Song, Shuang-Shuang Zhang, Shun-Fan Zhou, Jian-Xiang Liu

**Affiliations:** State Key Laboratory of Genetic Engineering, Collaborative Innovation Center for Genetics and Development, Institute of Plant Biology, School of Life Sciences, Fudan University, Shanghai, China; University of California Riverside, United States of America

## Abstract

The unfolded protein response (UPR) is activated to sustain cell survival by reducing misfolded protein accumulation in the endoplasmic reticulum (ER). The UPR also promotes programmed cell death (PCD) when the ER stress is severe; however, the underlying molecular mechanisms are less understood, especially in plants. Previously, two membrane-associated transcriptions factors (MTFs), bZIP28 and bZIP60, were identified as the key regulators for cell survival in the plant ER stress response. Here, we report the identification of another MTF, NAC089, as an important PCD regulator in Arabidopsis (*Arabidopsis thaliana*) plants. NAC089 relocates from the ER membrane to the nucleus under ER stress conditions. Inducible expression of a truncated form of NAC089, in which the transmembrane domain is deleted, induces PCD with increased caspase 3/7-like activity and DNA fragmentation. Knock-down *NAC089* in Arabidopsis confers ER stress tolerance and impairs ER-stress-induced caspase-like activity. Transcriptional regulation analysis and ChIP-qPCR reveal that NAC089 plays important role in regulating downstream genes involved in PCD, such as *NAC094*, *MC5* and *BAG6*. Furthermore, *NAC089* is up-regulated by ER stress, which is directly controlled by bZIP28 and bZIP60. These results show that nuclear relocation of NAC089 promotes ER-stress-induced PCD, and both pro-survival and pro-death signals are elicited by bZIP28 and bZIP60 during plant ER stress response.

## Author Summary

Protein folding is fundamentally important for development and responses to environmental stresses in eukaryotes. When excess misfolded proteins are accumulated in the endoplasmic reticulum (ER), the unfolded protein response (UPR) is triggered to promote cell survival through optimizing protein folding, and also promote programmed cell death (PCD) when the stress is severe. However, the link from ER-stress-sensing to PCD is largely unknown. Here, we report the identification of one membrane-associated transcription factor NAC089 as an important regulator of ER stress-induced PCD in plants. We have established a previously unrecognized molecular connection between ER stress sensors and PCD regulators. We have shown that organelle-to-organelle translocation of a transcription factor is important for its function in transcriptional regulation. Our results have provided novel insights into the molecular mechanisms of PCD in plants, especially under ER stress conditions.

## Introduction

In eukaryotic cells, ER is a major site for the production of secreted, plasma membrane and organelle proteins. Cells have evolved a sophisticated quality control system to ensure the accuracy of protein folding through optimizing the protein-folding machinery and ER-associated degradation (ERAD) [1], [2], [3]. To coordinate protein-folding capacity with protein-folding demand, a collection of phylogenetically conserved signaling pathways, termed the UPR, senses the accumulation of misfolded proteins in the ER and sustains homeostatic balance according to the protein folding needs which change constantly depending on different developmental programs and/or environmental conditions [1], [4], [5].

Three arms of UPR signaling pathways, namely inositol requiring enzyme 1 (IRE1), double-stranded RNA-activated protein kinase (PKR) like ER kinase (PERK), and activating transcription factor 6 (ATF6), were identified in mammalian cells that have the ability to promote cell survival by reducing misfolded protein accumulation in the ER. IRE1 is a key component in the most conserved branch, which acts by splicing messenger RNA encoding transcription factor Hac1p in yeast or XBP1 in mammalian cell, respectively [6], [7], [8]. Recently, the equivalent pathways were discovered in plants (e.g. the IRE1-bZIP60 pathway in Arabidopsis), which also play important roles in heat stress response, as well as in plant immune response [9], [10], [11], [12], [13], [14]. PERK is an ER-localized kinase and its activation upon ER stress leads to the attenuation of bulk protein translation in metazoan cells [15]. ATF6 is an ER membrane-associated bZIP transcription factor; its activation requires ER-to-Golgi translocation and regulated intramembrane proteolysis (RIP) [16]. Although the plant PERK ortholog has not yet been reported, the ER membrane-associated Arabidopsis bZIP28 was found to be the functional homolog of mammalian ATF6, which is activated in a manner similar to ATF6 [17], [18], [19], [20], [21].

Severe or chronic ER stress can also lead to PCD, a process that kills unwanted cells under ER stress conditions to protect other cells [22]. In contrast to what is known about how UPR protects cells, less is known about the mechanisms that link UPR to PCD, especially in plants [23]. In mammalian cells, IRE1 can trigger PCD by activating the Jun amino-terminal kinase (JNK) pathway [24]. Phosphorylation of JNK leads to the activation of pro-death protein BIM and inhibition of anti-death protein BCL-2 [25]. Mammalian IRE1 also binds to BAX and BAK, two cell-death-inducing proteins involved in the mitochondrial cell death pathway [26]. The activation of mammalian IRE1 is able to cause rapid decay of selected microRNAs (miRs -17, -34a, -96, and -125b) that normally repress translation of caspase-2 mRNA, and thus sharply elevates protein levels of this initiator protease in the mitochondrial cell death pathway [27]. The ER stress-induced mammalian bZIP transcription factor CHOP is one of the major players that induces PCD, most probably through suppression of the pro-survival protein BCL-2 and up-regulation of ERO1α to further perturb the cellular redox state [28]. CHOP is a downstream target of all three aforementioned UPR signaling pathways in mammals [4]. Recently, transcriptional induction through ATF4 and CHOP was shown to increase protein synthesis leading to oxidative stress and PCD [29]. Orthologs of mammalian JNK, BAX, BAK, CHOP and ATF4 are not found in the Arabidopsis genome [30]. However, ER stress-induced PCD is reported in plants with the hallmark of DNA segmentation, and the conserved BAX inhibitor-1 (BI-1) plays important roles in suppression of ER stress-induced PCD in Arabidopsis plant [31], [32], [33], [34], [35], [36], [37], [38]. When animal cells are subjected to severe ER stress, IRE1 loses its specificity and begins to degrade mRNAs in a process called regulated IRE1-dependent decay (RIDD) [39]. IRE1 in Arabidopsis also has similar function in the RIDD process in the UPR for degradation of mRNA encoding proteins in the secretory pathway to decrease the amount of proteins entering the ER [40]. Different from the animal system, knock-outs of both IRE1s in Arabidopsis impairs UPR and enhances PCD upon ER stress, indicating that RIDD may play a negative role in PCD in plants [40]. Despite the emerging evidence on ER stress-mediated PCD in plants, the underlying molecular mechanisms of PCD in plant UPR is still largely unknown. In soybean plants, prolonged ER stress and osmotic stress synergistically activate N-rich proteins (NRPs) to induce the expression of *NAC6/NAC30* to regulate PCD together with *NAC081* [33], [41], [42], however, the link from ER stress-sensing machinery to these NRPs is still missing.

Here we show that NAC089 plays important roles in regulating ER-stress-induced PCD in the model plant Arabidopsis. NAC089 relocates from the ER membrane to the nucleus during ER stress response. Inducible expression of a truncated form of NAC089 promotes PCD with increased caspase 3/7-like activity and DNA fragmentation. Down-regulation of *NAC089* confers ER stress tolerance and impairs ER-stress-induced caspase 3/7-like activity. Several UPR downstream genes including the PCD regulators *MC5*, *BAG6* and *NAC094* are shown to be regulated by NAC089 under ER stress condition. *NAC089* itself is also up-regulated by ER stress, which is directly controlled by bZIP28 and bZIP60. Therefore, NAC089 is an important PCD regulator in plant UPR, linking ER stress-sensing to downstream PCD regulators during ER stress response in plants.

## Results

### Up-regulation of *NAC089* by ER stress is controlled by both bZIP28 and bZIP60

**Figure 1:**
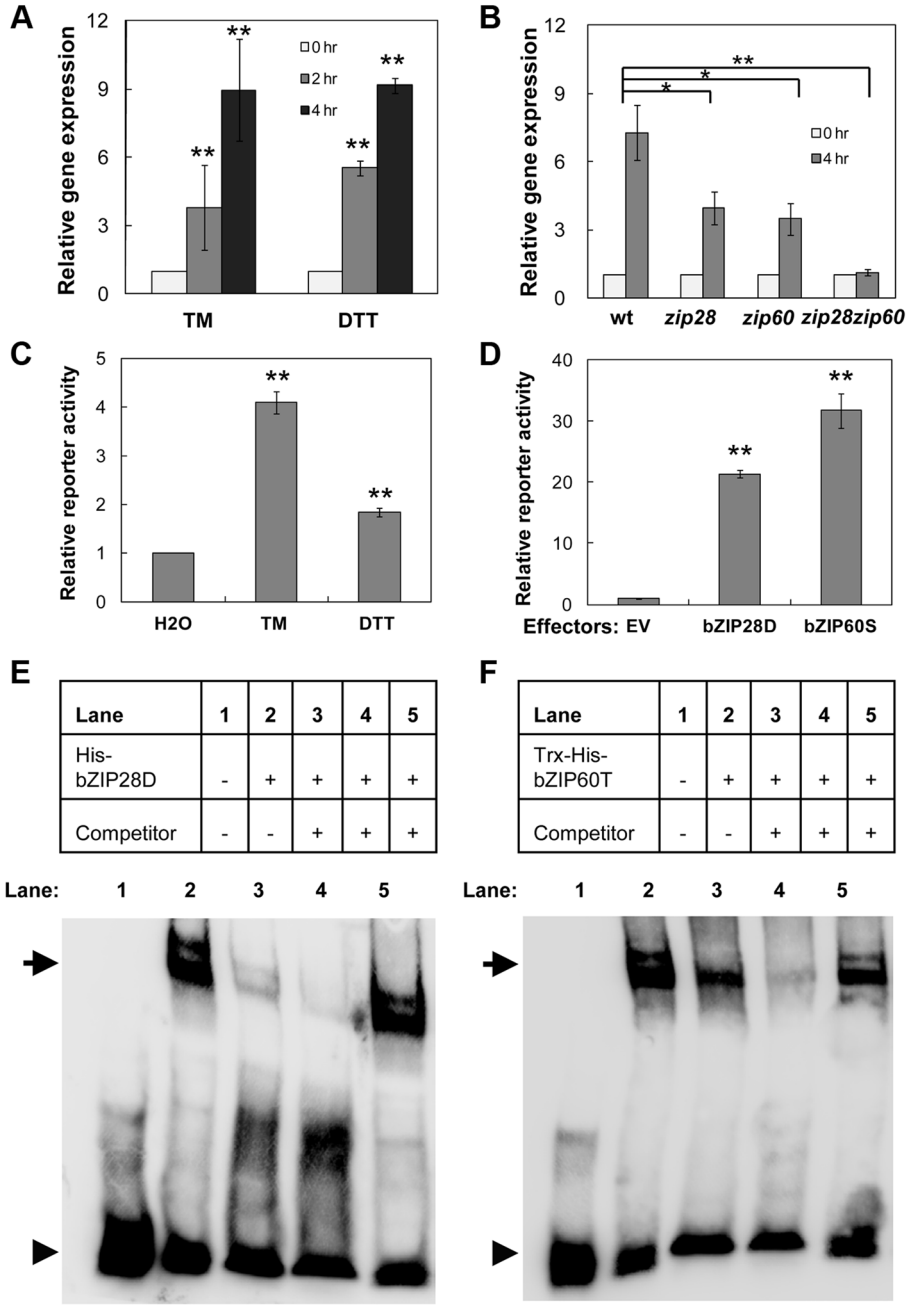
Up-regulation of *NAC089* by ER stress is directly controlled by bZIP28 and bZIP60. (A–B) Up-regulation of *NAC089* in the wild-type (wt) plants by ER-stress inducers tunicamycin (TM, 5 µg/ml) and dithiothreitol (DTT, 2 mM) (A) and in the wt, *bZIP28* single mutant (*zip28*), *bZIP60* single mutant (*zip60*) or double mutant (*zip28zip60*) by TM (5 µg/ml) treatment (B). The expression of *NAC089* is normalized to the expression of the internal control *actin*. (C–D) Transactivation of *NAC089* promoter in the dual-luciferase leaf protoplast assays. Activations of the *NAC089* promoter by ER stress treatments (C) and by co-expression of either activated bZIP28 (bZIP28D) or activated bZIP60 (bZIP60S) (D). Relative reporter activity is the firefly luciferase activity normalized to the renilla luciferase activity. Bars depict SE (n = 3) in A–D. The empty vector (EV) was used as a negative control. ** P<0.01, * P<0.05. (E–F) EMSA experiments to detect the protein-DNA binding. Either the purified His-bZIP28D (E) or Trx-His-bZIP60T (F) was incubated with the biotin-labeled pNAC089 DNA. Lane 3, 50× un-labeled pNAC089; lane 4, 200× un-labeled pNAC089; lane 5, 200× un-labeled mutated form pNAC089M1. Arrows and arrow heads point to the positions of shifted bands and free probes, respectively.

An ER-stress-related NAC (for NAM, ATAF, and CUC) transcription factor *NAC089* (also known as *ANAC089*) was identified from our previous microarray analysis [43]. Its expression was up-regulated rapidly by ER stress inducers tunicamycin (TM) and dithiothreitol (DTT) (Figure 1A and Figure S1). Knock-out of either *bZIP28* or *bZIP60* partially suppressed, while knock-outs of both *bZIP28* and *bZIP60* in *zip28zip60* double mutant completely abolished the up-regulation of *NAC089* under ER stress condition (Figure 1B). Previously two ER stress responsive *cis*-elements UPRE and ERSE-I were identified as the binding sites of bZIP28 and bZIP60 [21], [43], [44]. We searched the *NAC089* promoter region and found that one copy of UPRE and one copy of ERSE-I like (one mismatch) *cis*-elements are present over the segment [−95, −49] relative to the TSS site of *NAC089*. To assess the activation of *NAC089* promoter by bZIP28 and bZIP60, an effector-reporter dual-luciferase transient assay was set up. The *NAC089* promoter fragment containing the aforementioned *cis*-elements was fused to the firefly luciferase reporter and tested in Arabidopsis leaf protoplasts. As expected, the reporter was activated by either TM or DTT treatment (Figure 1C). Using this assay system, co-expression of either bZIP28D or bZIP60S dramatically enhanced the firefly luciferase reporter activity (Figure 1D). To demonstrate the direct binding of bZIP28 or bZIP60 to the *NAC089* promoter, electrophoretic mobility shift assays (EMSAs) were performed with the biotin-labeled *NAC089* promoter DNA. When the truncated form of either bZIP28 or bZIP60 was incubated with the biotin-labeled DNA probe, a band shift was observed reflecting the formation of the respective complex. To show the binding specificity, excess un-labeled probe was added and shown to be an effective competitor for the formation of each complex. On the contrary, the un-labeled mutated UPRE probe could not compete with the binding (Figure 1E–F). Through further mutation analysis, it was found that neither bZIP28 nor bZIP60 binds to the ERSE-I like *cis*-element presented in the probe (Figure S2). Thus, the expression of *NAC089* is up-regulated by ER stress, which is directly controlled by both bZIP28 and bZIP60 through the UPRE *cis*-element.

### NAC089 relocates from the ER membrane to the nucleus under ER stress condition

**Figure 2:**
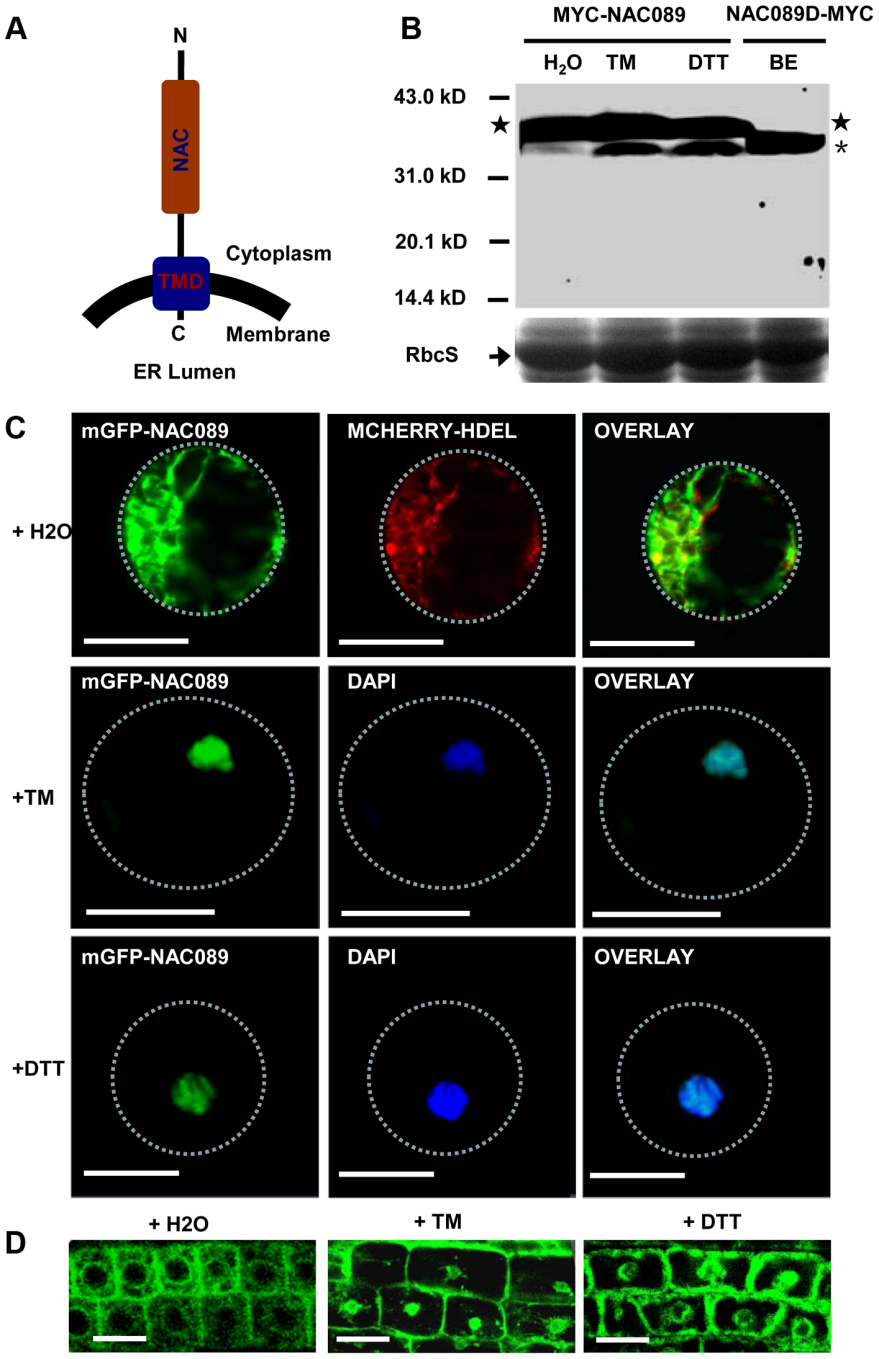
NAC089 relocates from the ER membrane to the nucleus during ER stress responses. (A) Topology of NAC089 protein. (B) Activation of MYC-NAC089 in response to ER stress. Plant seedlings were treated with H_2_O (control), tunicaymin (TM, 5 µg/ml) or DTT (2 mM) for 6 hr. Star and asterisk represent the precursor and the processed form, respectively. The beta-estradiol (BE) induced truncated form NAC089D-MYC was used as the migration marker. Coomassie blue staining of RbcS serves as the loading control. (C–D) Nuclear relocation of mGFP-NAC089 in response to ER stresses in Arabidopsis leaf protoplast (C) and root cells (D). TM = 5 µg/ml in C and TM = 10 µg/ml in D. Dashed lines highlight the cell boundary. Bar = 50 µm.

NAC089 is predicted to be a membrane-associated transcription factor [45] with the N-terminal DNA-binding domain facing the cytoplasm (Figure 2A). It has transcriptional activation activity and forms homodimers [46] (Figure S3). To confirm the membrane association of NAC089 and also to investigate the possible membrane-to-nucleus translocation of NAC089 in response to ER stress, 4X MYC tag was fused to NAC089 at the N-terminus and the fusion protein was expressed in Arabidopsis plants. Total proteins were extracted from transgenic seedlings and MYC-NAC089 was detected with the western blotting analysis. Without TM or DTT treatment, one prominent band reacted with the *anti*-MYC antibody. After TM or DTT treatment for 6 hr, one excess band with smaller molecular weight was induced, with a similar migration rate to the truncated form NAC089D, in which the C-terminal 24 amino acids of NAC089 were replaced with the 4X MYC tag (Figure 2B). To track the movement of NAC089 in response to ER stress, mGFP-NAC089 was expressed in Arabidopsis and observed under confocal laser scanning microscopy. In the mock (H_2_O) treatment, most of the mGFP-NAC089 signals were observed in the ER (Figure 2C–D); after either TM or DTT treatment for 6 hr, the fluorescence signals were largely found in the nuclei of the Arabidopsis leaf protoplasts and root cells (Figure 2C–D). The nuclear relocation of mGFP-NAC089 was not observed in the root cells when the transgenic plants were treated with TM (5 µg/ml) for short period of time (e.g. 2 hr). The ER-to-nucleus movement was also confirmed in the protein fractionation studies (Figure S4). Taken together, NAC089 is an ER membrane-associated protein and it relocates from the ER membrane to the nucleus in response to ER stress.

### Down-regulation of *NAC089* confers ER stress tolerance in plants

**Figure 3:**
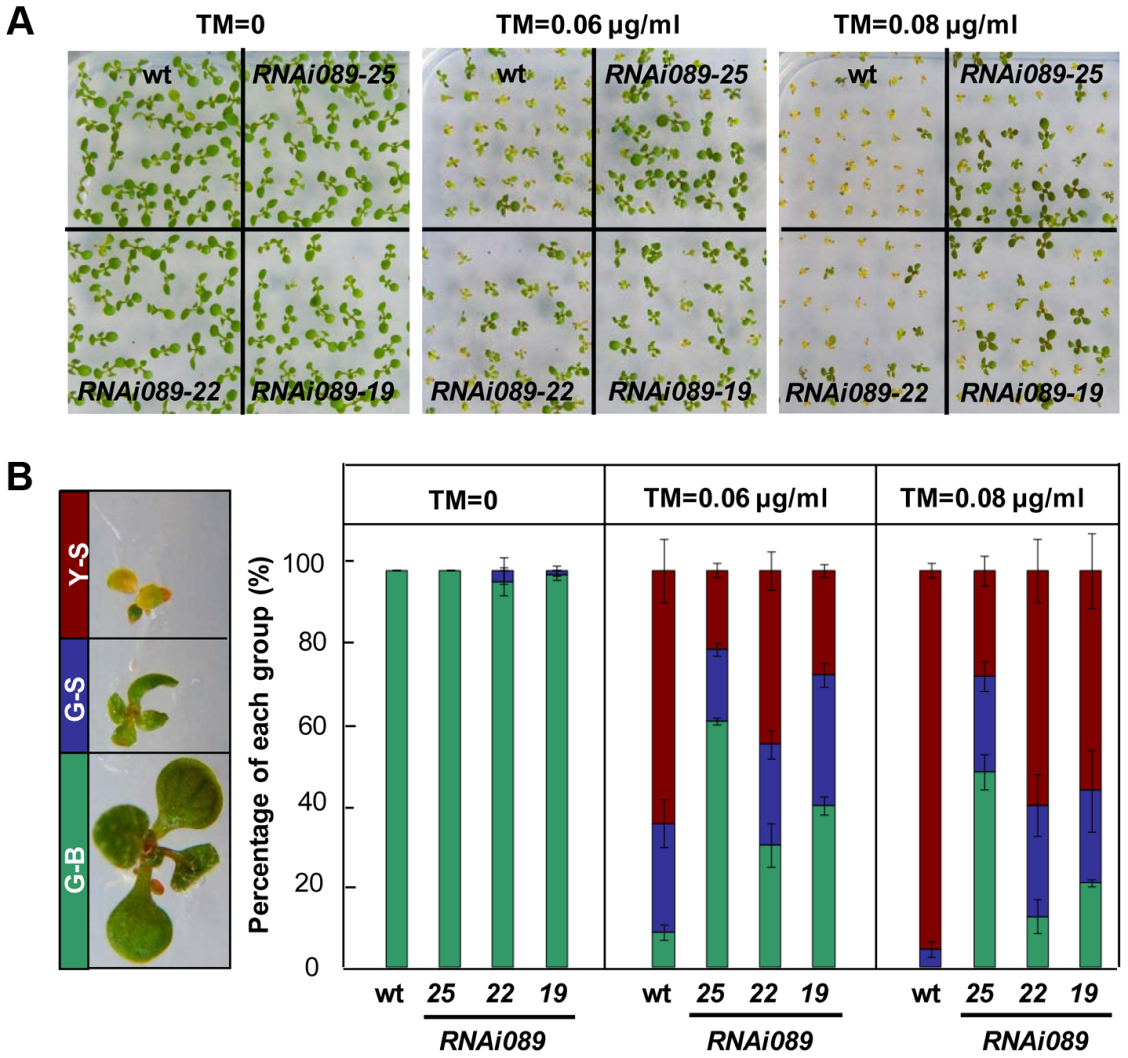
Down-regulation of *NAC089* confers ER stress tolerance. (A–B) Sensitivity of *NAC089* RNAi plants to ER stress. Photos were taken with 10-day-old Arabidopsis seedlings grown on 1/2 MS medium supplied without or with different concentrations of tunicamycin (TM) (A) and percentage of green-big (G-B), green-small (G-S) and yellow-small (Y-S) plants was calculated (B). Bars depict SE (n = 3). The difference between the wild-type (wt) and each *NAC089* RNAi line is significant (P<0.05).

To investigate the biological function of *NAC089* in the ER stress response, we created partial loss-of-function mutants by RNA interference (RNAi) and chimeric repressor silencing technology (CRES-T). *NAC089* knock-down plants (*RNAi089*) grew as well as the wild-type (wt) control under normal growth condition, but they were more tolerant to ER stress than the wt (Figure 3A–B, Figure S5). More greenish big (G-B) plants and less yellowish small (Y-S) plants were observed in the *RNAi089* plants than that in the wt under ER stress conditions (Figure 3B). In CRES-T system, fusion of an EAR-motif repression domain to a transcription factor converts an activator into a repressor, which results in partial loss function of the transcription factor [47]. We replaced the C-terminal hydrophobic tail of NAC089 with the EAR-motif, and expressed the chimerical fusion protein NAC089D-EAR in Arabidopsis with *NAC089*'s native promoter. NAC089D-EAR expression did not affect seedling development under normal growth condition, but also conferred ER stress tolerance in plants (Figure S6A–D). ER stress should be built-up in plants which had been grown on solid growth medium with low concentrations of tunicamycin for a long period of time, as ascertained by the up-regulation of UPR marker genes in the wild-type plants (Figure S6E). All together, we concluded that partial loss-of-function of *NAC089* in Arabidopsis increases chronic ER stress tolerance.

### NAC089 promotes programmed cell death in plants

**Figure 4:**
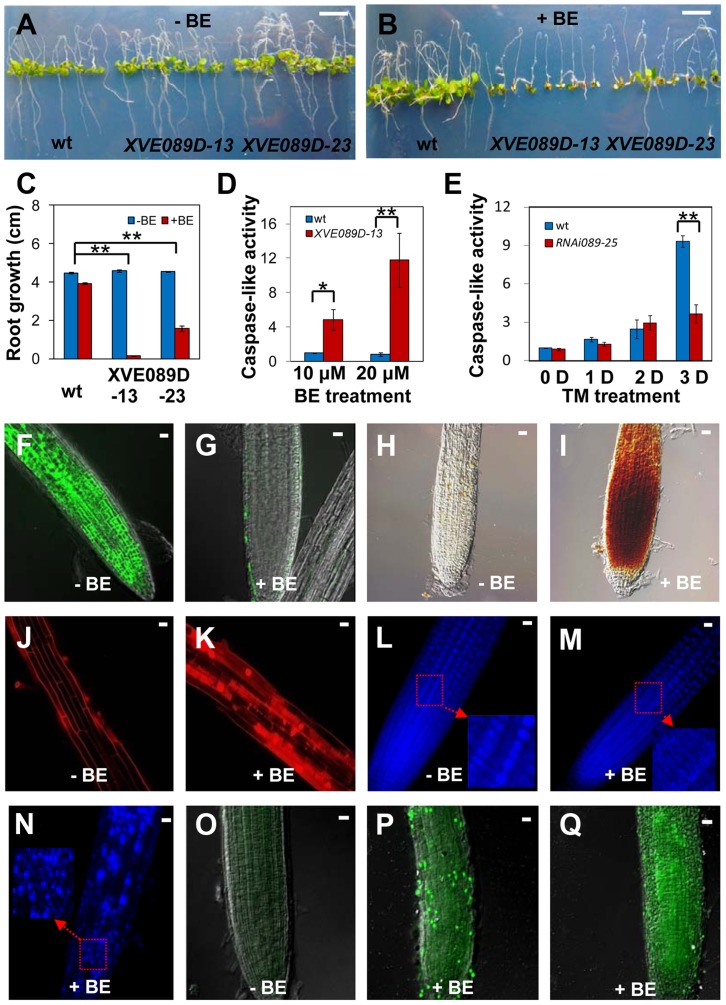
NAC089 promotes programmed cell death in plants. (A–C) Photos of Arabidopsis NAC089D-MYC transgenic lines (*XVE089D*) and wild-type (wt) control plants transferred to 1/2 MS medium supplied without (A) or with (B) beta-estradiol (BE) for additional 5 days with the quantitative data in (C). (D–E) Caspase 3/7-like activity in protein extracts from NAC089D-MYC overexpression plants treated with BE (D) or in the wt and *NAC089* RNAi plants treated with TM (0.5 µg/ml) (E). The caspase-like activity was normalized to the activity of the wt control under normal condition. Bars depict SE (n = 3) in C–E. ** P<0.01, * P<0.05. (F–I) Esterase activities (F–G) and H_2_O_2_ accumulation (H–I) in the *XVE089D-13* roots as revealed by FDA and DAB staining without (F, H) or with (G, I) BE treatment for 3 days. (J–N) Membrane rigidity and nucleus morphology in the *XVE089D-13* roots as reflected by PI (J–K) and DAPI (L–N) staining without (J, L) or with (K, M–N) BE treatment for 5 days. (O–Q) DNA breakage in the *XVE089D-13* roots as detected by TUNEL assay without (O) or with (P–Q) BE treatment for 5 days. Bar = 10 mm in A–B and bar = 10 µm in F–Q.

To gain insight into mechanisms by which NAC089 operates, we conditionally expressed a MYC-tagged truncated form of NAC089 (NAC089D-MYC) with the beta-estradiol (BE) inducible system [48] (Figure S7). This truncated form co-migrates with the ER stress-induced nuclear form of MYC-NAC089, has the transcriptional activation activity and localizes in the nucleus as mentioned above. The wt control and *NAC089D-MYC* expressing plants (*XVE089D*) were transferred to growth medium supplemented with or without BE. There was no obvious difference between the wt and *XVE089D* plants on the growth medium without BE (Figure 4A). However, when BE was included in the growth medium, root growth of the *XVE089D* plants was inhibited, and chlorotic leaves were observed, representing a typical PCD phenotype (Figure 4B–C). Cysteine-dependent aspartate-directed proteases (caspases) are the key regulators of PCD in animals, of which caspase-3 is the crucial executioner of PCD and recognize tetra-peptide motif DEVD [49]. Although the ortholog of animal caspase-3 is absent in plants, caspase 3/7-like activity has been reported in many examples involved in plant development and adaptation to environmental stresses [50]. To investigate whether the NAC089D-MYC-induced PCD was associated with the caspase-like activity, we performed caspase-3/7 activity assays using the same tetra-peptide substrate as previously reported [32], [33], [41]. It was found that the expression of *NAC089D-MYC* considerably induced caspase 3/7-like activity in the *XVE089D* plants (Figure 4D). The caspase 3/7 activity was also checked in the wt control and *NAC089* RNAi plants (line *RNAi089-25*). ER stress gradually induced caspase 3/7-like activity in both the wt and *RNAi089-25* plants in response to chronic ER stress. However, the caspase 3/7-like activity in the *RNAi089-25* plants was about half of that in the wt plants after 3 days of TM treatment (Figure 4E), suggesting that NAC089-regulated caspase 3/7-like activity is stress severity-dependent. It is possible that other pathways also regulate such caspase 3/7-like activity. Loss of cell viability, accumulation of H_2_O_2_ and rupture of plasma membrane are often associated with PCD [30]. To assess cell viability, roots of *XVE089D* plants were stained with fluorescein diacetate (FDA), which is a substrate for many endogenous esterases. *NAC089D-MYC* expression dramatically reduced the endogenous esterase activities (Figure 4F–G). Further 3, 3′-diaminobenzidine (DAB) staining demonstrated that H_2_O_2_ was accumulated in the roots when *NAC089D-MYC* was induced (Figure 4H–I). Propidium iodide (PI) binds to DNA, but it is often used to stain plant cell wall or plasma membrane (Figure 4J) because it is membrane impermeant. When *NAC089D-MYC* was induced, PI signals were observed in both the cytoplasm and nucleus (Figure 4K), indicating that expression of *NAC089D-MYC* reduced the rigidity of cell membrane. Another characteristic of PCD is the morphological changes in the nucleus which could be revealed by 4, 6′-diamidino-2-phenylindole (DAPI) staining. Intact and round nuclei were found in most of the *XVE089D* root cells without BE treatment (Figure 4L). In contrast, nuclei with stretches and speckles were observed in the *XVE089D* root cells when the plants were treated with BE (Figure 4M–N). Cleavage of genomic DNA at internucleosomal sites by endogenous nucleases is always associated with PCD and terminal deoxynucleotidyl transferase-mediated dUTP nick and labeling (TUNEL) assay is frequently used to label the fragmentation of nuclear DNA *in situ* [31]. Compared to the low level of background green fluorescence in normal-grown roots (Figure 4O), strong TUNEL-positive signals were observed in the *XVE089D* root cells when the plants were treated with BE (Figure 4P–Q). BE treatment had no obvious effect on the aforementioned histochemical staining in the wt control plants (Figure S8). These results suggest that NAC089 has the ability to promote PCD in plants.

### *NAC089* regulates many downstream genes including PCD regulators under ER stress condition

**Figure 5:**
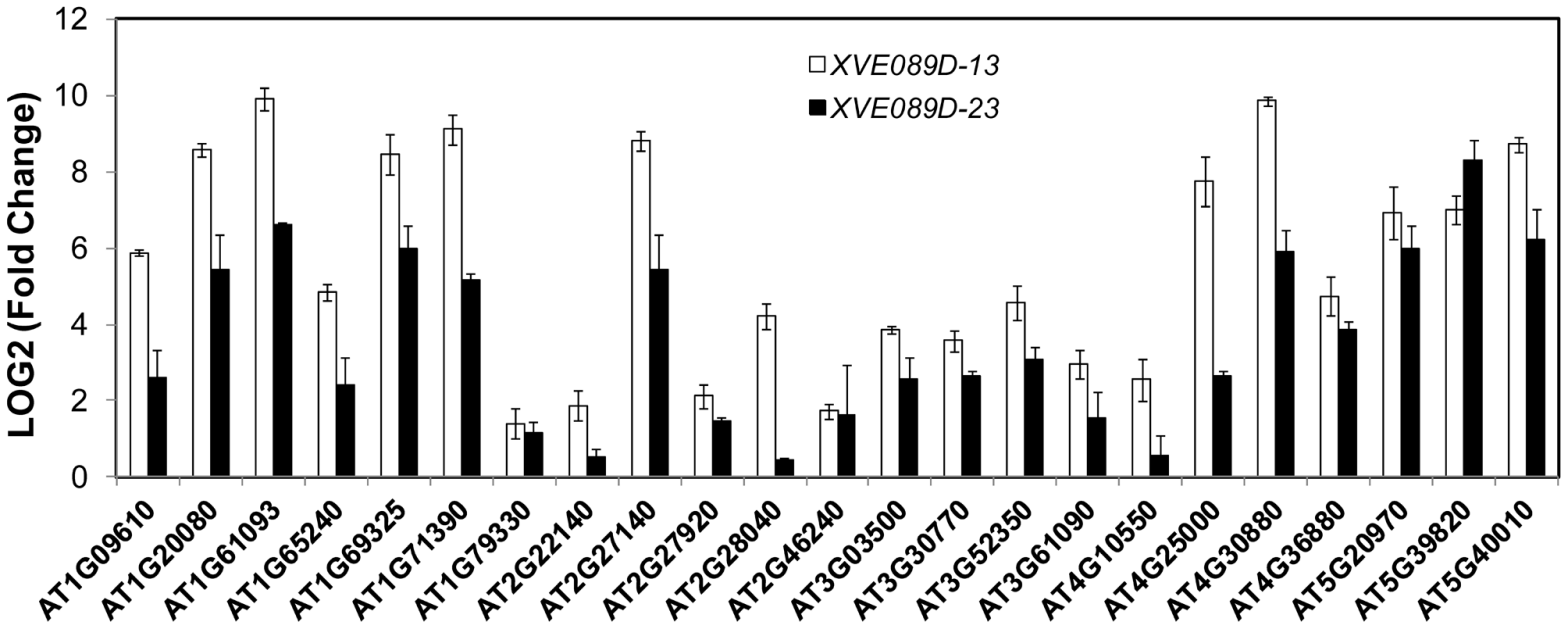
NAC089 regulates many downstream genes. Expression of down-stream genes identified through microarray analysis was examined with qRT-PCR in two lines of *NAC089D-MYC* plants. Fold change is the gene expression level of plants treated with beta-estradiol (BE) for 16 hr divided by that of plants treated with DMSO (solvent control), both of which were normalized to the expression of *actin*. Bars depict SE (n = 3). The fold change for each gene is significant (P<0.01) in line *XVE089D-13*.

**Figure 6:**
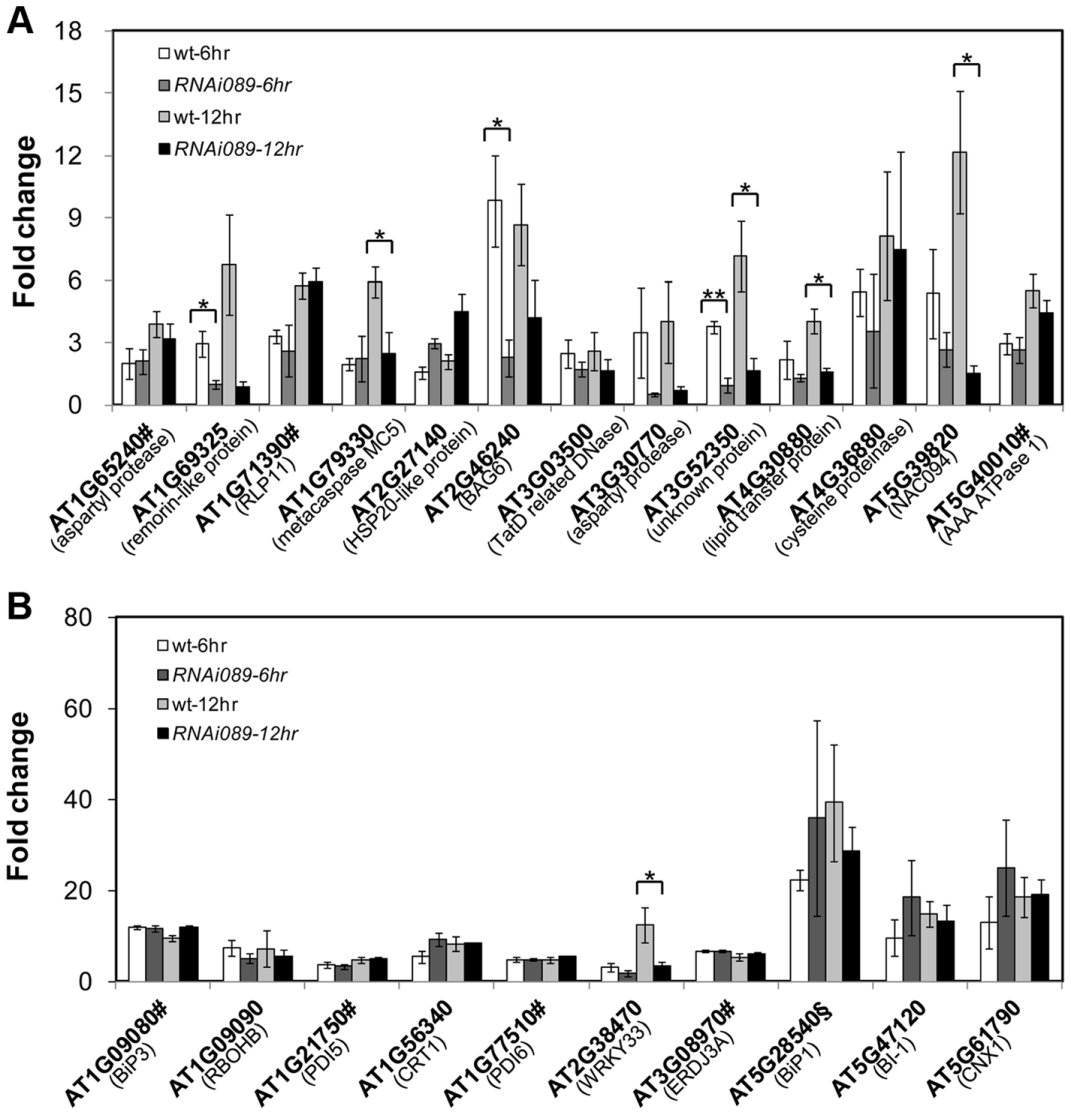
Up-regulation of NAC089D-MYC-regulated genes by ER stress is impaired in the *NAC089* RNAi plants. The expressions of NAC089D-MYC downstream genes (A) and other UPR markers (B) were quantified with qRT-PCR in the wild-type control (wt) and *NAC089* knock-down plants (line *RNAi089-25*). Fold change is the gene expression value in TM (5 µg/ml) -treated plants divided by the value in non-treated plants, both of which were normalized to the expression of *actin*. Bars depict SE (n = 3). Values for the gene locus with # are log2 values. Fold change of BiP1 (AT5g28540, highlighted with §) also includes that of BiP2 (AT5G42020). ** P<0.01, * P<0.05.

**Figure 7:**
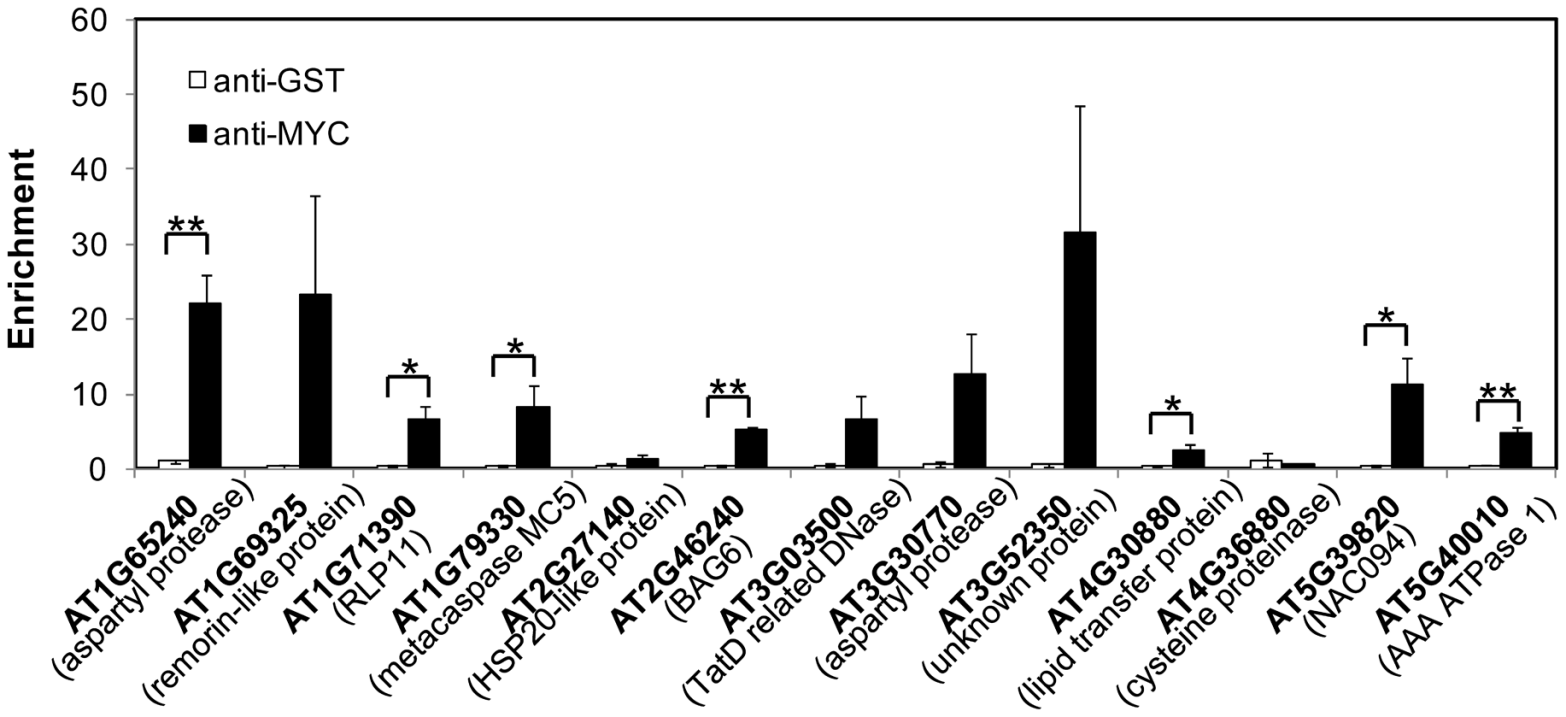
NAC089 binds to the promoter regions of target genes. Enrichment is the DNA level of each fragment in beta-estradiol (BE)-treated sample divided by that in DMSO-treated sample, both of which were normalized to the level of *TA3*. *Anti*-MYC antibody was used to precipitate NAC089D-MYC and *anti*-GST was used as a negative antibody control. Bars depict SE (n = 3). ** P<0.01, * P<0.05 except for AT1G79330 (p = 0.057).

PCD is a genetically controlled process that plays important roles in plant development and responses to abiotic stress or pathogens [51], [52]. Many PCD regulators that have been well characterized in humans, worms and flies are absent from the Arabidopsis genome, indicating that plants may use different regulators to execute PCD [52], [53]. To understand how NAC089 regulates plant PCD, we performed microarray (Agilent 4X44K) experiments with the BE inducible gene expression system [48]. BE treatment did not affect much of the gene expression in the wt plants as reported by other colleagues [54] (Figure S9), but up-regulated 1363 probes (fold change >2, P<0.01) in the *NAC089D-MYC* expressing plants (*XVE089D-13*) (Dataset S1). Gene ontology (GO) analysis revealed that the most significant GO term among the NAC089D-MYC-regulated genes is the transcription factor activity (Figure S10), indicating that NAC089 is an important transcriptional regulator. To validate the microarray expression data, 23 genes were selected from the microarray data and their expression was examined by qRT-PCR in two *NAC089D-MYC* expressing lines. It was found that the expression of these genes was highly induced in both transgenic lines, especially in line *XVE089D-13* (Figure 5). Among the 23 selected genes, 13 of them were up-regulated more than two fold by the prolonged ER stress, especially after 12 hr TM treatment (Figure S11). The up-regulation of these 13 genes by ER stress was also checked in the wt and *NAC089* knock-down plants (line *RNAi089-25*). Previously, the BAX inhibitor 1 (BI-1) was reported to be an important modulator of ER stress-mediated PCD in Arabidopsis [31]. The transcription factor WRKY33, which is required for resistance to necrotrophic pathogens, plays critical roles in autophagy [55]. These two PCD markers along with other cell survival UPR markers were also included in the expression study. It was found that the up-regulation of 6 NAC089D-MYC-regulated genes (i.e. *AT1G69325*, encoding remorin-like protein; *AT1G79330*, encoding metacaspase MC5; *AT2G46240*, encoding BCL-2-associated athanogene BAG6; *AT3G52350*, encoding unknown protein; *AT4G30880*, encoding lipid transfer protein; and *AT5G39820*, encoding transcription factor NAC094) and autophagy-related gene *WRKY33* was impaired in the *RNAi089-25* plants under ER stress condition comparing to the wt control (Figure 6A–B). These results indicate that *NAC089* plays critical roles in regulating these ER-stress-induced genes including several PCD-related genes. ER stress also up-regulates several cell survival UPR marker genes and BI-1 in both the wt and *RNAi089-25* plants (Figure 6B), suggesting that NAC089 plays minor role in regulating the expression of these genes. In order to know whether *NAC089* directly regulates these downstream genes, chromatin immunoprecipitation (ChIP) experiments were carried out with NAC089D-MYC plants (line *XVE089D-13*) using *anti*-MYC antibody. It was found that NAC089D-MYC was enriched significantly with fold change greater than 2 at the promoter regions of 7 genes (i.e. *AT1G65240*, *AT1G71390*, *AT1G79330*, *AT2G46240*, *AT4G30880*, *AT5G39820* and *AT5G40010*) (Figure 7), indicating that these genes might be the direct targets of NAC089. Among the 7 NAC089 possible targets, the up-regulation of 3 genes (i.e. *AT1G65240*, encoding aspartyl protease; *AT1G71390*, encoding receptor-like protein RLP11 and *AT5G40010*, encoding AAA ATPase 1) by ER stress was not suppressed in the *NAC089* knock-down mutants (Figure 6A), suggesting that other factors may also up-regulate these genes under ER stress condition. We concluded that NAC089 has the ability to regulate some of the UPR downstream genes, including the PCD regulatory genes *MC5*, *BAG6* and *NAC094*, and the autophagy regulatory gene *WRKY33*. The function of other NAC089 downstream genes in ER stress-induced PCD needs to be investigated in the future.

## Discussion

The unmitigated ER stress is believed to induce PCD in animals [3], as well as in plants [31], [56]. Given that PCD components are not highly conserved between animals and plants [30], [57], our knowledge on ER stress-induced PCD in plants is very limited [58]. Previously, BI-1 and IRE1 were reported to be the negative regulators of PCD in plants [31], [40]. In the current study, a membrane-associated transcription factor NAC089 was identified as an important transcriptional regulator of plant PCD under ER stress condition based on the following evidences: 1) *NAC089* is up-regulated by UPR regulators bZIP28 and bZIP60 under ER stress condition; 2) NAC089 relocates from the ER membrane to the nucleus in response to ER stress; 3) Inducible expression of the truncated form of NAC089 induces PCD; 4) Partial loss-of-function of *NAC089* confers resistance to chronic ER stress with reduced caspase 3/7-like activity; 5) NAC089 has transcriptional activity and binds to the promoter of many downstream targets; 6) Knock-down *NAC089* suppresses the ER-stress-induced expression of several PCD regulators.

As in animals, plant development and adaptations to environmental stresses are intimately connected to PCD [59], [60]. In mammals, PCD is controlled predominately through functionally conserved proteins such as CED9/BCL-2 and BAX, but such genes have not been identified in plants [52]. Interestingly, the heterotrimeric G protein signaling was reported to be involved in ER stress-associated PCD. Null mutants of G beta subunit (*AGB1*) were more resistant to ER stress than either the wt plants or null mutants of G alpha subunit, but the underlying molecular mechanism was not known yet [61]. On the contrary, Chen and Brandizzi recently reported that the null *AGB1* mutants were more sensitive to ER stress [62]. The function of AGB1 in ER stresses response needs to be further clarified. Caspases are cysteine-aspartic proteases that play essential roles in PCD in animals [4]. Plant caspase homologs are not found so far, and the metacaspases were demonstrated to have similar function in plant PCD [53], [63], [64]. Caspase-like activity has been detected in plant PCD associated with xylem formation and adaptations to heavy metal stress, pathogen infection, as well as exposure to ultraviolent-C [50], [65], [66], [67], [68]. In the current study, chronic ER stress induced caspase 3/7-like activity, and such induction was impaired in the *NAC089* knock-down plants. Several NAC089 downstream targets including some known PCD regulators were also indentified in the current study. Among them, one metacaspase (*MC5*) and several other proteases were induced by ER stress, which was suppressed in the *NAC089* knock-down plants. BAG (BCL2-associated athanogene) family proteins were originally identified as the anti-cell-death protein in mammals [69]. Among the seven animal BAG homologs found in Arabidopsis [70], overexpression of *BAG6* induced PCD in yeast and plants [71], indicating that BAG6 is a pro-death protein in plants. In the current study, *BAG6* was induced by ER stress in the Arabidopsis wt plants, which was impaired in the *NAC089* knock-down plants. Previously, the soybean NAC transcription factor *NAC6/NAC30* was shown to induce caspase 3-like activity and promote extensive DNA fragmentation when it was overexpressed in soybean protoplasts [33]. Here in the current study, we found that one of the direct targets of *NAC089*, *NAC094*, is the close-related homolog of soybean *NAC6/NAC30* in Arabidopsis. ER stress-induced expression of *NAC094* was greatly suppressed in the *NAC089* knock-down plants. These results support that NAC089 controls the expression of several PCD-related downstream genes in Arabidopsis under ER stress condition. Interestingly, the autophagy-related gene *WRKY33* [55]was also up-regulated by ER stress, which was dependent on *NAC089*. Other NAC089 downstream genes such as genes encoding protease and nuclease were also identified in the current study. The identification of NAC089 as a PCD regulator provides more opportunities for further understanding new molecular components involved in plant PCD, especially under ER stress condition.

NAC089 is regulated by ER stress at both transcriptional and post-translational levels. At the transcriptional level, *NAC089* is up-regulated by ER stress, which is directly controlled by bZIP28 and bZIP60, two important regulators in plant UPR [9], [18], [44], [72]. At the protein level, NAC089 is an ER membrane-associated transcription factor (MTF) and it relocates from the ER to the nucleus under ER stress condition. Interestingly, bZIP28 and bZIP60 are also ER MTFs. bZIP28 is activated through regulated proteolysis. In response to ER stress, bZIP28 relocates from the ER to the Golgi where it is cleaved by two Golgi-resident proteases S1P and S2P, and the C-terminal lumen-facing domain is thought to be responsible for the sensing of ER stress [17], [18], [19], [72], [73]. The activation mechanism for bZIP60 is unconventional, and the activation of bZIP60 is dependent on the ER membrane-localized IRE1 proteins. Under ER stress conditions, *bZIP60* mRNA is spliced by IRE1, which results in an open reading frame (ORF) shift and elimination of the transmembrane domain [9], [11], [12]. The N-terminal part of yeast IRE1 is inserted into the ER lumen and plays important role in direct sensing the unfolded proteins in the ER in yeast [74]. Recently, at least 13 MTFs in NAC family are found in Arabidopsis, of which some are activated during development and adaptations to environmental stresses [45], [75], [76], [77], [78]. However, the activation mechanisms of these NAC MTFs are still largely unknown. The *NAC089* mRNA does not have the predicted double stem-loop structure that has been shown to be very important for IRE1 splicing [9]. Furthermore, there is no alternative spliced transcript of *NAC089* observed in the ER stressed wt seedlings (Figure S12), suggesting that NAC089 might be activated in a manner different from bZIP60. The C-terminal ER lumen facing tail of NAC089 is very short and does not have the canonical S1P cutting site, which implicates that NAC089 might not be proteolytically processed in the same way as bZIP28. We did not include protease inhibitors in the NAC089 activation experiments because most of the protease inhibitors are not permeable to live plant cells. Further investigation of the activation mechanism of NAC089 will improve our understanding of MTFs in plants. Surprisingly, one rare nucleotide polymorphism caused by natural variation in the Arabidopsis *Cvi* ecotype results in premature stop and constitutive nuclear localization of NAC089, in which the C-terminus (114 AA) including the hydrophobic tail is not translated. Although *Cvi* ecotype is much more sensitive to fructose than *Ler* ecotype, expressing the *Cvi NAC089* suppresses fructose sensitivity in *Ler* seedlings [79]. The truncated form (114 AA deletions) of NAC089 was found in the nucleus, however, the activation or nuclear relocation of NAC089 in response to fructose treatment is not reported, and how the truncated form of NAC089 represses fructose signaling is not clear. Since the deletion occurred in *Cvi* was found neither in over 100 Arabidopsis accessions nor in the Arabidopsis Genome 1001 sequence collections, the biological function of NAC089 in fructose signaling other than in the *Cvi* ecotype is elusive [79]. Recently, it was reported that fructose feeding induced ER stress in mice [80]. It would be interesting to determine whether the high concentration of fructose could also induce ER stress in Arabidopsis plant. Recently, NAC089 was reported to be involved in redox regulation [81]. Besides its effect on redox status, DTT also inhibits disulphide bond formation and therefore promotes protein misfolding. However, TM is a more specific ER stress inducer because of its specific effect on blocking protein N-glycosylation in the ER. In the current study, both TM and DTT treatments were employed to demonstrate the specific role of NAC089 in ER stress response. Our current study has also advanced the understanding of the function of NAC089 in ER-stress-induced plant PCD and the underlying molecular mechanisms.

How cells make the cell fate selection between life and death remains enigmatic. In human cells, ER stress activates all the three arms of UPR pathways; each branch has different effect on cell survival or cell death, but attenuation of each branch is different. Switch between cell survival and cell death outputs lies in part in the duration of individual branch activity, which guides the cell toward survival or demise [82]. In plants, except the PERK pathway, the bZIP28 and bZIP60 branches of signaling pathway have been previously discovered to regulate downstream genes involved in promoting cell survival [83]. Knock-outs of both branches in the *zip28zip60* double mutant causes high sensitivity to ER stresses and accelerated PCD under prolonged ER stress condition [72]. IRE1A and IRE1B redundantly control the activation of bZIP60 and RIDD; knock-outs of both *IRE1*s in Arabidopsis also promotes PCD while knock-out of single *bZIP60* gene has no PCD phenotype [40]. The ER stress-induced up-regulation of *NAC089* is dependent on both *bZIP28* and *bZIP60*. Different from the *zip28zip60* mutant, knock-down *NAC089* confers ER stress tolerance and over-expression of the truncated form of NAC089 promotes PCD. These results may not necessarily be controversial. Firstly, bZIP28 and bZIP60 regulate many survival genes whose expressions are almost completely abolished in the *zip28zip60* mutant [72]. Lacking the expression of survival genes in the *zip28zip60* mutant may lead to the accelerated PCD. Secondly, *NAC089* has substantial constitutive expression and the NAC089 pathway may still operate for PCD in the *zip28zip60* mutant even without further *NAC089* up-regulation. Thirdly, it is possible to have other PCD pathways turned on to execute PCD in the *zip28zip60* mutant. Recently, heterotrimeric G protein signaling [61], vacuolar processing enzyme (VPE)-triggered cell death [42], [84] and IRE1-mediated autophagy [85] pathways were reported to be involved in the ER stress-induced PCD in plants.

**Figure 8:**
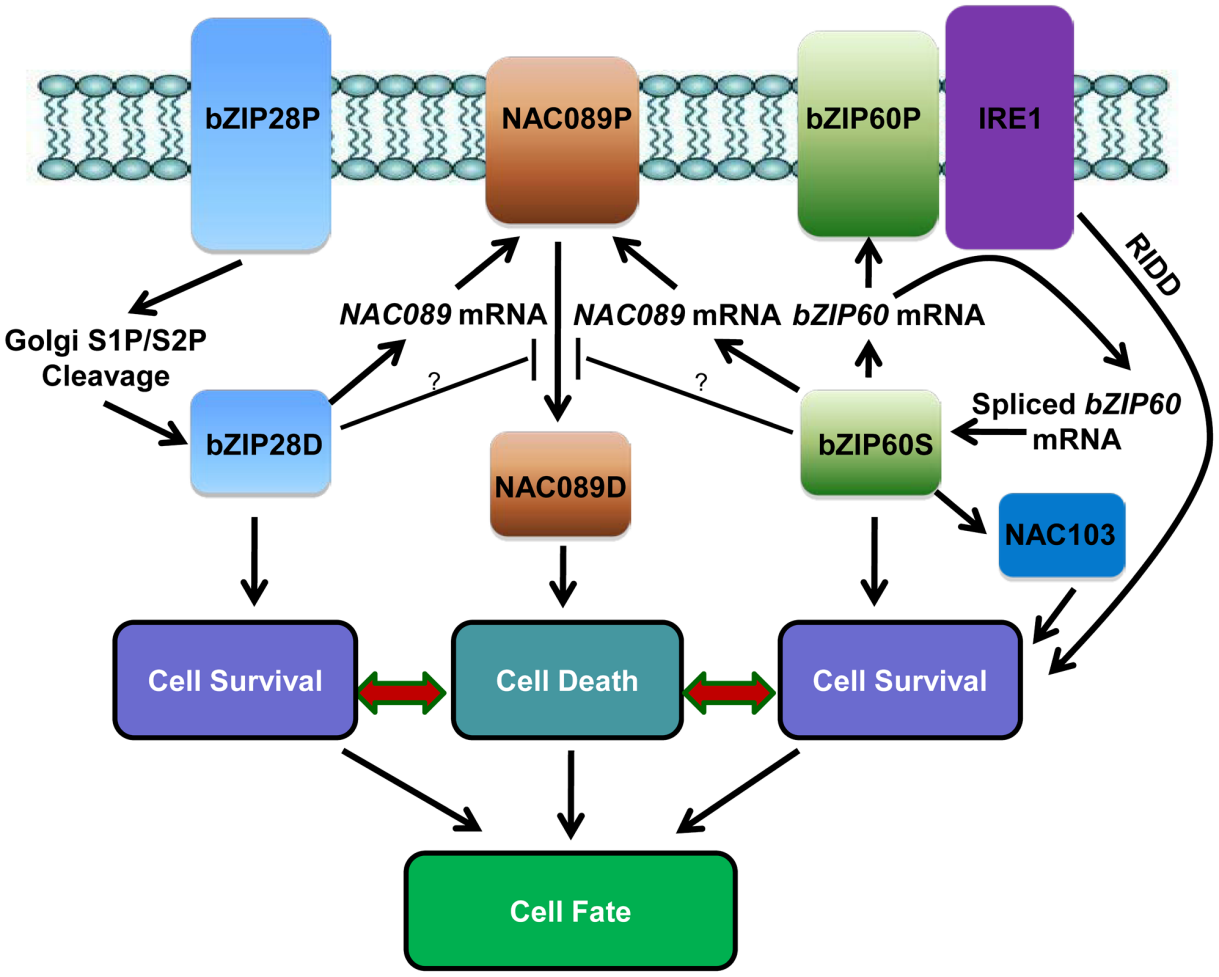
Hypothetical working model of cell survival and cell death pathways in plant UPR. In response to ER stress, the ER-localized bZIP28P is activated through S1P/S2P sequential cleavage. The precursor bZIP60P is also ER membrane-associated under normal growth condition; ER stress activates the ER-localized IRE1, which splices the *bZIP60* mRNA to produce a shorter *bZIP60* mRNA encoding the activated form bZIP60S. Both the activated bZIP28D and bZIP60S enter the nucleus and up-regulate downstream genes such as molecular chaperones and ERAD components to ensure cell survival. The activated bZIP60S also induces its own transcription and another transcription factor *NAC103* to amplify the cell survival signal. IRE1 could also protect cells through RIDD to reduce the entry of secretory proteins into the ER lumen. On the other hand, the ER membrane-localized NAC089P is activated to produce the nuclear form NAC089D when the ER stress is severe. NAC089D activates several downstream PCD-related genes to promote cell death. Both bZIP28D and bZIP60S control the up-regulation of *NAC089* under ER stress condition and may also control the expression of negative regulators for NAC089 activation. Thus, both pro-survival and pro-death signals are controlled by bZIP28 and bZIP60, and the balance between their outputs probably decides the life-or-death cell fate in Arabidopsis plants under ER stress conditions.

A hypothetical working model has been emerged from the current study (Figure 8). When Arabidopsis cells are confronted with ER stress, both bZIP28 and bZIP60 pathways are activated to mitigate the stress by up-regulation of genes involved in protein folding or ERAD to improve survival [1], [83]. The activated bZIP60 also induces its own transcription [44] and another transcription factor *NAC103* [86] to amplify the cell survival signal. The ER-localized IRE1 protects cell through a process called RIDD to reduce the protein folding demand [40]. In the meantime, besides the constitutive high expression of *NAC089* under normal condition, both bZIP28 and bZIP60 up-regulate the expression of *NAC089* under ER stress condition. Up-regulation of *NAC089* mRNA may increase the protein level of the membrane-associated NAC089 precursor. However, nuclear relocation of NAC089 is tightly controlled, in which bZIP28 and bZIP60 may play negative roles. When the ER stress is severe, NAC089 is activated and relocates from the ER to the nucleus, inducing the expression of PCD regulators to promote cell death. It is possible that the stress intensity and/or duration of ER stress might determine the signaling output and the final cell fate. Further understanding on how cells balance the cell survival and cell death effects in UPR is of great fascination.

## Materials and Methods

### Plant materials, growth conditions and phenotypic analysis

All Arabidopsis (*Arabidopsis thaliana*) wild-type, T-DNA mutants and transgenic plants in the current study were in the Columbia (Col-0) ecotype background. The double mutant *zip28zip60* was made as previously reported [72]. Methods for plant growth were described previously [43]. For short time treatment, different concentrations of TM (5 µg/ml), DTT (2 mM) or BE (10 µM) were added in the half-strength MS medium unless mentioned in the text. For long time treatment, 10 µM BE or various low concentrations of TM were supplied in the solid growth medium. Root length was measured and emergence rate of true leaves was calculated. Total chlorophylls were extracted from seedlings with 80% (v/v) acetone at 4°C overnight and measured from A_663_ and A_646_ readings for each sample [87]. All the data in the paper were subjected to Student's *t*-test or two-way ANOVA (analysis of variance) analysis.

### Plasmid construction and generation of transgenic plants

The coding sequence of *NAC089* was amplified with PCR and inserted into pSKM36 after digestion with *Asc*I and *Spe*I restriction enzymes to produce the vector Pro35S:NAC089. Modified green fluorescence protein (mGFP) tag and 4X MYC tag were amplified and inserted into Pro35S:NAC089 at *Asc*I site to generate the Pro35S:mGFP-NAC089 and Pro35S:MYC-NAC089 constructs, respectively. For RNAi construct preparation, part of the *NAC089* gene sequence covering cDNA 651–1150 was inserted into pHANNIBAL in both sense and antisense orientations separated by an intron sequence. The entire RNAi cassette was cut with *Not*I and inserted into pART27 to make the RNAi expression vector. To express the dominant negative fusion protein ProNAC089:NAC089D-EAR, the sequence encoding EAR motif (QDLDLELRLGFA) was synthesized and firstly inserted into pCAMBIA1300; about 1 kb upstream sequence of *NAC089* and sequence encoding the truncated form of NAC089 (NAC089D, aa 1–316) were amplified and subsequently inserted. To generate the conditionally overexpression construct [48], nucleotides encoding NAC089D was amplified and inserted into pER10M. For dual luciferase activity assay, fragment of the *NAC089* promoter (−98 bp to −46 bp relative to the TSS site) was synthesized and inserted into pGreen0800-II after the 35S minimal promoter was introduced. Constructs expressing bZIP28D and bZIP60S were made as described [9], [18]. For protein expression in *E. coli*, the respective sequence of bZIP18D (aa 1–321) or bZIP60T (aa 87–217) was amplified and inserted into pET28 and pET32, respectively. All the primers were listed in Table S1 and error-free clones were introduced into plants by either transient expression or stable transformation.

### Protein extraction and immunoblotting analysis

Total protein was extracted from plants with extraction buffer described previously [17]. Membrane fraction and nuclear fraction were prepared with sucrose gradient centrifuge according to the standard protocol [88]. Proteins were resolved on 8–10% SDS-PAGE gels and visualized by western blotting using antibodies against c-MYC (Santa Cruz Biotechnology), nuclear protein marker histone H3 (Abcam) and ER protein marker BiP (Santa Cruz Biotechnology).

### Caspase-like activity and luciferase reporter assays

Caspase-like activity was measured with luminescent assays based on DEVD short peptides with Caspase-Glo 3/7 Assay Kit (Promega). Both caspase 3 and caspase 7 recognize the same DEVD substrate. Briefly, seedlings were harvested after various treatments and total proteins were extracted with liquid nitrogen in a buffer containing 100 mM sodium acetate, pH 5.5, 100 mM NaCl, 1 mM EDTA, and 5 mM DTT. To measure caspase-3/7 activity, 30 µl caspase-3/7 luminogenic substrate (Z-DEVD-aminoluciferin) was added to 50 µg protein extracts and incubated at 22°C for 1 hr protected from light. The luminescence of each sample was measured with the Synergy 2 Multi-Mode Microplate Reader (BioTek). For dual-luciferase activity assays [89], Arabidopsis leaf protoplasts were isolated from 4-week-old soil-grown seedlings and transfected according to a standard protocol [43] with various reporter constructs or cotranstransfected with different effectors. Firefly and renilla luciferase were quantified with Dual-Luciferase Reporter Assay Kit (Promega) according to the manufacturer's instructions in the Synergy 2 Multi-Mode Microplate Reader (BioTek).

### Histochemistry and microscopy

For FDA and DAB staining, seedlings were stained with 2.5 µg/ml FDA (Sigma-Aldrich) in phosphate-buffered saline for 10 min or 1 mg/ml DAB (pH 5.5, Sigma-Aldrich) for 2 hr at room temperature, immersed into boiled ethanol for 10 min according to the standard protocol [31]. For PI and DAPI staining, samples were stained using DAPI (Sigma-Aldrich) at 1 µg/ml in 0.1% (v/v) Triton X-100 for 10 min or PI (Sigma-Aldrich) at 10 µg/ml for 1 min, and washed twice with water. For *in situ* TUNEL staining, roots were stained in microcentrifuge tubes (1.5 ml) using the *in situ* cell death detection kit (Takara) according to the manufacturer' instructions. Except DAB staining, which was observed under differential interference contrast (DIC) microscopy, other staining, BiFC and subcellular localization of mGFP-NAC089 were visualize with laser confocal fluorescence microscopy (Zeiss LSM A710).

### Chromatin immunoprecipitation (ChIP) and gene expression analysis

ChIP was performed according to the standard protocols. Briefly about 3 g of 2-week-old *XVE089D* transgenic seedlings were treated with either 10 µM BE or DMSO (solvent control) for 16 hr and fixed with 1.0% formaldehyde for 10 min subsequently. Antibodies against c-MYC (Santa Cruz Biotechnology) and GST (IgG control, Abmart) were used for immunoprecipitation. Protein-A-agarose beads were blocked with salmon sperm DNA and used to pull down the protein-DNA complex. Equal amounts of starting plant material and the ChIP products were used for quantitative PCR. Primers were selected in the promoter regions of each selected gene. DNA levels were calculated relative to *TA3* (AT1G37110) using a comparative threshold cycle method. The ChIP experiments were performed 3 times with biological replications and similar results were obtained. For microarray analysis or qRT-PCR, the wt control, *XVE089D* and *NAC089* RNAi plants were grown vertically on agar plates for one week and then transferred to 1/2 MS liquid supplied with 10 µM BE or DMSO or TM for a period of time as noted. Total RNA was extracted and purified according to the manufacturer's instructions [43]. Agilent Arabidopsis gene chips (4X44K) were used to compare the gene expression profiles with three independent replications. P-values were calculated and used to select the genes that are up-regulated by NAC089D-MYC (cut-off: P<0.01, fold change >2). Microarray data from this article can be found in ArrayExpress under the accession number E-MTAB-1377. Quantitative PCR and RT-PCR were routinely conducted [43] and all the primers are listed in Table S1. GO analysis was performed with AgriGO (http://bioinfo.cau.edu.cn/agriGO/analysis.php).

### Electrophoretic mobility shift assay (EMSA)

EMSA was performed using a LightShift Chemiluminescent EMSA Kit (Pierce), according to the manufacturer's protocols [43]. Briefly, each 20 µL binding reaction contained 2 µl binding buffer, 0.3 µl Poly (dI-dC), 4 µg purified protein, 0.83 µmol biotin-labeled probe or certain amount of unlabeled probe as the competitor. The pNAC089 wt or pNAC089M1-M3 probes were created by annealing together complementing oligonucleotides and biotinylated with a labeling kit (Pierce). His-tagged bZIP28D or Trx-His-tagged bZIP60T proteins were expressed in *E. coli* strain BL21 and purified with Ni-NTA agarose beads (Qiagen). The binding reactions were allowed to incubate on ice for 1 hr and run on a 5% polyacrylamide mini-gel (37.5∶1 acrylamide-bisacrylamide in 0.5× Tris-Borate-EDTA (TBE) containing 3% glycerol). The complex was transferred to a membrane and developed according to a standard protocol.

## Supporting Information

Dataset S1 List of genes regulated by conditional overexpression of *NAC089D-MYC*.
(XLS)

Figure S1 *NAC089* is up-regulated by ER stress. (A–B) Up-regulation of *NAC089* by tunicamycin (TM, A) and dithiothreitol (DTT, B) was examined in the dose-response experiments. The expression of *NAC089* is normalized to the expression of the internal control *actin*. The wild-type Arabidopsis seedlings were stressed for 4 hr. Bars depict SE (n = 3).
(TIF)

Figure S2 bZIP28 and bZIP60 bind to UPRE but not to ERSE-I-like *cis*-element. (A) DNA sequences of biotin-labeled probes. The predicted ER stress responsive *cis*-elements UPRE and ERSE-I-Like were highlighted in bold. The mutated sites were underlined. (B–C) EMSA for protein and DNA interactions. Either the purified His-bZIP28D (B) or Trx-His-bZIP60T (C) was incubated with various biotin-labeled DNA. Arrows and arrow heads point to the positions of shifted bands and free probes, respectively.
(TIF)

Figure S3 NAC089 has transcriptional activation activity and forms homodimmers. (A) Schematic structure of NAC089 protein. DBD: DNA binding domain; TMD: transmembrane domain. (B) Transcriptional activation activity of NAC089. Segment of NAC089 lacking the TMD (NAC089D) was fused to yeast GAL4 DNA binding domain and the activation of *HIS* and *LacZ* reporters were evaluated. (C–F) Evaluation of dimmer formation in yeast 2-hybrid assays (C–D) and BiFC assays (E–F). Bar = 50 µm.
(TIF)

Figure S4 NAC089 is enriched in the membrane fraction or nuclear fraction depending on different conditions. Plant seedlings were treated with H_2_O (control), TM or DTT for 6 hr and the used for protein fractionation studies. *Anti*-Histone and *anti*-BiP antibodies were employed to detect the nuclear protein marker Histone H3 and ER protein marker BiP, respectively. The beta-estradiol (BE) induced truncated form NAC089D-MYC was used as the migration marker.
(TIF)

Figure S5 *NAC089* is specifically knocked-down in the RNAi plants. (A–B) The expression of *NAC089* (A) and its close-related homologs *NAC060* and *NAC040* (B) in the *NAC089* knock-down plants. The relative gene expression in the transgenic plants is the value normalized to the expression in the wild-type control (wt), both of which are normalized to the internal control *actin*. Bars depict SE (n = 3).
(TIF)

Figure S6 Partial loss-of-function of NAC089 enhances ER stress tolerance. (A–D) The C-terminal hydrophobic region of NAC089 was replaced by the transcriptional repressor domain EAR and the fusion protein was validated by RT-PCR in Arabidopsis plants (A). Photos of 12-day-old Arabidopsis grown on 1/2 MS medium supplied without or with different concentration of TM were taken (B). Emergence rate of true leaves was counted (C) and total chlorophyll content of seedlings was measured (D). The wild-type (wt) and *zip28zip60* double mutant were used as the controls. Bars depict SE (n = 3). (E) Expression of UPR marker genes in the wild-type plants after growing on 1/2 MS medium supplied without or with different concentration of TM for 12 days. *UBQ5* was used as an internal control.
(TIF)

Figure S7 NAC089D-MYC is expressed in the overexpression plants. Validation of transgenic expression in two lines of *NAC089D-MYC* overexpression plants by western blotting. Coomassie blue staining of RbcS serves as a loading control for western blotting. Plant seedlings were treated with beta-estradiol (BE) for 16 hr.
(TIF)

Figure S8 Beta estradiol has little effect on histochemical staining in the wild-type control plants. (A–D) Esterase activities (A–B) and H_2_O_2_ accumulation (C–D) in the wild-type control roots as revealed by FDA and DAB staining without (A, C) or with (B, D) beta-estradiol (BE) treatment for 3 days. (E–H) Membrane rigidity and nucleus diffusion in the wild-type control roots as reflected by PI (E–F) and DAPI (G–H) staining without (E, G) or with (F, H) BE treatment for 5 days. (I–J) DNA breakage in the wild-type control roots as detected by TUNEL assay without (I) or with (J) BE treatment for 5 days. Bar = 10 µm.
(TIF)

Figure S9 Beta estradiol does not induce *NAC089* downstream genes in the wild-type control plants. Wild-type control (wt) and *XVE089D-MYC* plants were treated with DMSO (control) or beta-estradiol (BE) for 16 hr and gene expressions were checked with RT-PCR. *UBQ5* was used as an internal control
(TIF)

Figure S10 Transcription factor activity is enriched in the *NAC089* downstream genes. Gene Ontology (GO) analysis was performed with *NAC089D-MYC* induced genes in agriGO.
(TIF)

Figure S11 Some of the NAC089D-MYC-regulated genes are up-regulated by ER stress. Totally 23 genes were selected from the microarray experiment and their expressions were examined with qRT-PCR. The wild-type plants were treated with tunicamycin for 4 and 12 hr and the expression of NAC089 target genes was also quantified with qRT-PCR. The relative gene expression is the value in the treated sample normalized to the untreated control, both of which are normalized to the expression of *actin*. Bars depict SE (n = 3).
(TIF)

Figure S12 *NAC089* is not alternatively spliced under ER stress condition. (A) Gene model of *NAC089*. White rectangles represent UTRs and black rectangles denote exons. The region encoding the transmembrane domain of NAC089 is boxed. (B) Detection of *NAC089* transcript with RT-PCR. The wild-type plants were treated with H_2_0 (control), 5 µg/ml tunicamycin (TM) or 2 mM DTT for 4 hr and the expression of *NAC089* was examined by RT-PCR with different primer pairs. *UBQ5* was used as a loading control.
(TIF)

Table S1 Primers used in the study.
(DOC)

## References

[1] Liu JX, Howell SH (2010) Endoplasmic reticulum protein quality control and its relationship to environmental stress responses in plants. Plant Cell 22: 2930–2942. 20876830 10.1105/tpc.110.078154 PMC2965551

[2] Smith MH, Ploegh HL, Weissman JS (2011) Road to ruin: Targeting proteins for degradation in the endoplasmic reticulum. Science 334: 1086–1090. 22116878 10.1126/science.1209235 PMC3864754

[3] Walter P, Ron D (2011) The unfolded protein response: From stress pathway to homeostatic regulation. Science 334: 1081–1086. 22116877 10.1126/science.1209038

[4] Ron D, Walter P (2007) Signal integration in the endoplasmic reticulum unfolded protein response. Nat Rev Mol Cell Biol 8: 519–529. 17565364 10.1038/nrm2199

[5] Vitale A, Boston RS (2008) Endoplasmic reticulum quality control and the unfolded protein response: Insights from plants. Traffic 9: 1581–1588. 18557840 10.1111/j.1600-0854.2008.00780.x

[6] Cox JS, Shamu CE, Walter P (1993) Transcriptional induction of genes encoding endoplasmic-reticulum resident proteins requires a transmembrane protein-kinase. Cell 73: 1197–1206. 8513503 10.1016/0092-8674(93)90648-a

[7] Shen XH, Ellis RE, Lee K, Liu CY, Yang K, et al (2001) Complementary signaling pathways regulate the unfolded protein response and are required for C-elegans development. Cell 107: 893–903. 11779465 10.1016/s0092-8674(01)00612-2

[8] Calfon M, Zeng HQ, Urano F, Till JH, Hubbard SR, et al (2002) IRE1 couples endoplasmic reticulum load to secretory capacity by processing the XBP-1 mRNA. Nature 415: 92–96. 11780124 10.1038/415092a

[9] Deng Y, Humbert S, Liu JX, Srivastava R, Rothstein SJ, et al (2011) Heat induces the splicing by IRE1 of a mRNA encoding a transcription factor involved in the unfolded protein response in Arabidopsis. Proc Natl Acad Sci USA 108: 7247–7252. 21482766 10.1073/pnas.1102117108 PMC3084119

[10] Lu SJ, Yang ZT, Sun L, Sun L, Song ZT, et al (2012) Conservation of IRE1-regulated bZIP74 mRNA unconventional splicing in rice (Oryza sativa L.) involved in ER stress responses. Mol Plant 5: 504–514. 22199238 10.1093/mp/ssr115

[11] Nagashima Y, Mishiba KI, Suzuki E, Shimada Y, Iwata Y, et al (2011) Arabidopsis IRE1 catalyses unconventional splicing of bZIP60 mRNA to produce the active transcription factor. Sci Rep 1: 29. 22355548 10.1038/srep00029 PMC3216516

[12] Moreno AA, Mukhtar MS, Blanco F, Boatwright JL, Moreno I, et al (2012) IRE1/bZIP60-mediated unfolded protein response plays distinct roles in plant immunity and abiotic stress responses. PloS One 7: e31944. 22359644 10.1371/journal.pone.0031944 PMC3281089

[13] Hayashi S, Wakasa Y, Takahashi H, Kawakatsu T, Takaiwa F (2012) Signal transduction by IRE1-mediated splicing of bZIP50 and other stress sensors in the endoplasmic reticulum stress response of rice. Plant J 69: 946–956. 22050533 10.1111/j.1365-313X.2011.04844.x

[14] Deng Y, Srivastava R, Howell SH (2013) Protein kinase and ribonuclease domains of IRE1 confer stress tolerance, vegetative growth, and reproductive development in Arabidopsis. Proc Natl Acad Sci USA 110: 19633–19638. 24145452 10.1073/pnas.1314749110 PMC3845099

[15] Harding HP, Zhang YH, Ron D (1999) Protein translation and folding are coupled by an endoplasmic-reticulum-resident kinase. Nature 397: 271–274. 9930704 10.1038/16729

[16] Haze K, Yoshida H, Yanagi H, Yura T, Mori K (1999) Mammalian transcription factor ATF6 is synthesized as a transmembrane protein and activated by proteolysis in response to endoplasmic reticulum stress. Mol Biol Cell 10: 3787–3799. 10564271 10.1091/mbc.10.11.3787 PMC25679

[17] Liu JX, Srivastava R, Che P, Howell SH (2007) Salt stress responses in Arabidopsis utilize a signal transduction pathway related to endoplasmic reticulum stress signaling. Plant J 51: 897–909. 17662035 10.1111/j.1365-313X.2007.03195.x PMC2156172

[18] Liu JX, Srivastava R, Che P, Howell SH (2007) An endoplasmic reticulum stress response in Arabidopsis is mediated by proteolytic processing and nuclear relocation of a membrane-associated transcription factor, bZIP28. Plant Cell 19: 4111–4119. 18156219 10.1105/tpc.106.050021 PMC2217655

[19] Che P, Bussell JD, Zhou WX, Estavillo GM, Pogson BJ, et al (2010) Signaling from the endoplasmic reticulum activates brassinosteroid signaling and promotes acclimation to stress in Arabidopsis. Sci Signal 3 (141) ra69. 20876872 10.1126/scisignal.2001140

[20] Gao H, Brandizzi F, Benning C, Larkin RM (2008) A membrane-tethered transcription factor defines a branch of the heat stress response in Arabidopsis thaliana. Proc Natl Acad Sci USA 105: 16398–16403. 18849477 10.1073/pnas.0808463105 PMC2571009

[21] Tajima H, Iwata Y, Iwano M, Takayama S, Koizumi N (2008) Identification of an Arabidopsis transmembrane bZIP transcription factor involved in the endoplasmic reticulum stress response. Biochem Biophy Res Commun 374: 242–247. 10.1016/j.bbrc.2008.07.021 18634751

[22] Sitia R, Braakman I (2003) Quality control in the endoplasmic reticulum protein factory. Nature 426: 891–894. 14685249 10.1038/nature02262

[23] Tabas I, Ron D (2011) Integrating the mechanisms of apoptosis induced by endoplasmic reticulum stress. Nat Cell Biol 13: 184–190. 21364565 10.1038/ncb0311-184 PMC3107571

[24] Urano F, Wang XZ, Bertolotti A, Zhang YH, Chung P, et al (2000) Coupling of stress in the ER to activation of JNK protein kinases by transmembrane protein kinase IRE1. Science 287: 664–666. 10650002 10.1126/science.287.5453.664

[25] Shore GC, Papa FR, Oakes SA (2011) Signaling cell death from the endoplasmic reticulum stress response. Curr Opin Cell Biol 23: 143–149. 21146390 10.1016/j.ceb.2010.11.003 PMC3078187

[26] Hetz C, Bernasconi P, Fisher J, Lee AH, Bassik MC, et al (2006) Proapoptotic BAX and BAK modulate the unfolded protein response by a direct interaction with IRE1 alpha. Science 312: 572–576. 16645094 10.1126/science.1123480

[27] Upton JP, Wang L, Han D, Wang ES, Huskey NE, et al (2012) IRE1 alpha cleaves select microRNAs during ER stress to derepress translation of proapoptotic caspase-2. Science 338: 818–822. 23042294 10.1126/science.1226191 PMC3742121

[28] McCullough KD, Martindale JL, Klotz LO, Aw TY, Holbrook NJ (2001) Gadd153 sensitizes cells to endoplasmic reticulum stress by down-regulating Bc12 and perturbing the cellular redox state. Mol Cell Biol 21: 1249–1259. 11158311 10.1128/MCB.21.4.1249-1259.2001 PMC99578

[29] Han J, Backa SH, Hur J, Lin Y-H, Gildersleeve R, et al (2013) ER-stress-induced transcriptional regulation increases protein synthesis leading to cell death. Nat Cell Biol 15: 481. 23624402 10.1038/ncb2738 PMC3692270

[30] van Doorn WG, Beers EP, Dangl JL, Franklin-Tong VE, Gallois P, et al (2011) Morphological classification of plant cell deaths. Cell Death Differ 18: 1241–1246. 21494263 10.1038/cdd.2011.36 PMC3172093

[31] Watanabe N, Lam E (2008) BAX inhibitor-1 modulates endoplasmic reticulum stress-mediated programmed cell death in Arabidopsis. J Biol Chem 283: 3200–3210. 18039663 10.1074/jbc.M706659200

[32] Zuppini A, Navazio L, Mariani P (2004) Endoplasmic reticulum stress-induced programmed cell death in soybean cells. J Cell Sci 117: 2591–2598. 15159454 10.1242/jcs.01126

[33] Faria JAQA, Reis PAB, Reis MTB, Rosado GL, Pinheiro GL, et al (2011) The NAC domain-containing protein, GmNAC6, is a downstream component of the ER stress- and osmotic stress-induced NRP-mediated cell-death signaling pathway. BMC Plant Biology 11: 129. 21943253 10.1186/1471-2229-11-129 PMC3193034

[34] Alves MS, Reis PAB, Dadalto SP, Faria JAQA, Fontes EPB, et al (2011) A novel transcription factor, ERD15 (Early Responsive to Dehydration 15), connects endoplasmic reticulum stress with an osmotic stress-induced cell death signal. J Biol Chem 286: 20020–20030. 21482825 10.1074/jbc.M111.233494 PMC3103375

[35] Ishikawa T, Watanabe N, Nagano M, Kawai-Yamada M, Lam E (2011) Bax inhibitor-1: a highly conserved endoplasmic reticulum-resident cell death suppressor. Cell Death Differ 18: 1271–1278. 21597463 10.1038/cdd.2011.59 PMC3172100

[36] Kawai-Yamada M, Jin LH, Yoshinaga K, Hirata A, Uchimiya H (2001) Mammalian Bax-induced plant cell death can be down-regulated by overexpression of Arabidopsis Bax Inhibitor-1 (AtBl-1). Proc Natl Acad Sci USA 98: 12295–12300. 11593047 10.1073/pnas.211423998 PMC59808

[37] Kawai-Yamada M, Ohori Y, Uchimiya H (2004) Dissection of Arabidopsis Bax inhibitor-1 suppressing Bax-, hydrogen peroxide-, and salicylic acid-induced cell death. Plant Cell 16: 21–32. 14671021 10.1105/tpc.014613 PMC301392

[38] Sanchez P, Zabala MD, Grant M (2000) AtBI-1, a plant homologue of Bax Inhibitor-1, suppresses Bax-induced cell death in yeast and is rapidly upregulated during wounding and pathogen challenge. Plant J 21: 393–399. 10758491 10.1046/j.1365-313x.2000.00690.x

[39] Hollien J, Lin JH, Li H, Stevens N, Walter P, et al (2009) Regulated Ire1-dependent decay of messenger RNAs in mammalian cells. J Cell Biol 186: 323–331. 19651891 10.1083/jcb.200903014 PMC2728407

[40] Mishiba KI, Nagashima Y, Suzuki E, Hayashi N, Ogata Y, et al (2013) Defects in IRE1 enhance cell death and fail to degrade mRNAs encoding secretory pathway proteins in the Arabidopsis unfolded protein response. Proc Natl Acad Sci USA 110: 5713–5718. 23509268 10.1073/pnas.1219047110 PMC3619347

[41] Costa MDL, Reis PAB, Valente MAS, Irsigler AST, Carvalho CM, et al (2008) A new branch of endoplasmic reticulum stress signaling and the osmotic signal converge on plant-specific asparagine-rich proteins to promote cell death. J Biol Chem 283: 20209–20219. 18490446 10.1074/jbc.M802654200

[42] Mendes GC, Reis PAB, Calil IP, Carvalho HH, Aragao FJL, et al (2013) GmNAC30 and GmNAC81 integrate the endoplasmic reticulum stress- and osmotic stress-induced cell death responses through a vacuolar processing enzyme. Proc Natl Acad Sci USA 110: 19627–19632. 24145438 10.1073/pnas.1311729110 PMC3845183

[43] Liu JX, Howell SH (2010) bZIP28 and NF-Y transcription factors are activated by ER Stress and assemble into a transcriptional complex to regulate stress response genes in Arabidopsis. Plant Cell 22: 782–796. 20207753 10.1105/tpc.109.072173 PMC2861475

[44] Iwata Y, Koizumi N (2005) An Arabidopsis transcription factor, AtbZIP60, regulates the endoplasmic reticulum stress response in a manner unique to plants. Proc Natl Acad Sci USA 102: 5280–5285. 15781873 10.1073/pnas.0408941102 PMC555978

[45] Kim SY, Kim SG, Kim YS, Seo PJ, Bae M, et al (2007) Exploring membrane-associated NAC transcription factors in Arabidopsis: implications for membrane biology in genome regulation. Nucl Acid Res 35: 203–213. 10.1093/nar/gkl1068 PMC1802569 17158162

[46] Li J, Zhang J, Wang X, Chen J (2010) A membrane-tethered transcription factor ANAC089 negatively regulates floral initiation in Arabidopsis thaliana. Sci China-Life Sci 53: 1299–1306. 21046321 10.1007/s11427-010-4085-2

[47] Hiratsu K, Matsui K, Koyama T, Ohme-Takagi M (2003) Dominant repression of target genes by chimeric repressors that include the EAR motif, a repression domain, in Arabidopsis. Plant J 34: 733–739. 12787253 10.1046/j.1365-313x.2003.01759.x

[48] Zuo JR, Niu QW, Chua NH (2000) An estrogen receptor-based transactivator XVE mediates highly inducible gene expression in transgenic plants. Plant J 24: 265–273. 11069700 10.1046/j.1365-313x.2000.00868.x

[49] Porter AG, Janicke RU (1999) Emerging roles of caspase-3 in apoptosis. Cell Death Differ 6: 99–104. 10200555 10.1038/sj.cdd.4400476

[50] Han JJ, Lin W, Oda Y, Cui KM, Fukuda H, et al (2012) The proteasome is responsible for caspase-3-like activity during xylem development. Plant J 72: 129–141. 22680239 10.1111/j.1365-313X.2012.05070.x

[51] Kuriyama H, Fukuda H (2002) Developmental programmed cell death in plants. Curr Opin Plant Biol 5: 568–573. 12393021 10.1016/s1369-5266(02)00305-9

[52] Lam E, Kato N, Lawton M (2001) Programmed cell death, mitochondria and the plant hypersensitive response. Nature 411: 848–853. 11459068 10.1038/35081184

[53] Coll NS, Vercammen D, Smidler A, Clover C, Van Breusegem F, et al (2010) Arabidopsis type I metacaspases control cell death. Science 330: 1393–1397. 21097903 10.1126/science.1194980

[54] Ohashi-Ito K, Oda Y, Fukuda H (2010) Arabidopsis VASCULAR-RELATED NAC-DOMAIN6 directly regulates the genes that govern programmed cell death and secondary wall formation during xylem differentiation. Plant Cell 22: 3461–3473. 20952636 10.1105/tpc.110.075036 PMC2990123

[55] Lai Z, Wang F, Zheng Z, Fan B, Chen Z (2011) A critical role of autophagy in plant resistance to necrotrophic fungal pathogens. Plant J 66: 953–968. 21395886 10.1111/j.1365-313X.2011.04553.x

[56] Crosti P, Malerba M, Bianchetti R (2001) Tunicamycin and Brefeldin A induce in plant cells a programmed cell death showing apoptotic features. Protoplasma 216: 31–38. 11732194 10.1007/BF02680128

[57] Hoeberichts FA, Woltering EJ (2003) Multiple mediators of plant programmed cell death: interplay of conserved cell death mechanisms and plant-specific regulators. Bioessays 25: 47–57. 12508282 10.1002/bies.10175

[58] Eichmann R, Schafer P (2012) The endoplasmic reticulum in plant immunity and cell death. Front Plant Sci 3: 200–200. 22936941 10.3389/fpls.2012.00200 PMC3424470

[59] Cacas JL (2010) Devil inside: does plant programmed cell death involve the endomembrane system? Plant Cell Environ 33: 1453–1473. 20082668 10.1111/j.1365-3040.2010.02117.x

[60] Love AJ, Milner JJ, Sadanandom A (2008) Timing is everything: regulatory overlap in plant cell death. Trend Plant Sci 13: 589–595. 10.1016/j.tplants.2008.08.006 18824399

[61] Wang S, Narendra S, Fedoroff N (2007) Heterotrimeric G protein signaling in the Arabidopsis unfolded protein response. Proc Natl Acad Sci USA 104: 3817–3822. 17360436 10.1073/pnas.0611735104 PMC1820667

[62] Chen Y, Brandizzi F (2012) AtIRE1A/AtIRE1B and AGB1 independently control two essential unfolded protein response pathways in Arabidopsis. Plant J 69: 266–277. 21914012 10.1111/j.1365-313X.2011.04788.x

[63] Tsiatsiani L, Van Breusegem F, Gallois P, Zavialov A, Lam E, et al (2011) Metacaspases. Cell Death Differ 18: 1279–1288. 21597462 10.1038/cdd.2011.66 PMC3172103

[64] Lam E, Zhang Y (2012) Regulating the reapers: activating metacaspases for programmed cell death. Trend Plant Sci 17: 487–494. 10.1016/j.tplants.2012.05.003 22658651

[65] Ye Y, Li Z, Xing D (2013) Nitric oxide promotes MPK6-mediated caspase-3-like activation in cadmium-induced Arabidopsis thaliana programmed cell death. Plant Cell Environ 36: 1–15. 22621159 10.1111/j.1365-3040.2012.02543.x

[66] Zhang L, Xu Q, Xing D, Gao C, Xiong H (2009) Real-time detection of caspase-3-like protease activation in vivo using fluorescence resonance energy transfer during plant programmed cell death Induced by ultraviolet C overexposure. Plant Physiol 150: 1773–1783. 19535476 10.1104/pp.108.125625 PMC2719143

[67] Danon A, Rotari VI, Gordon A, Mailhac N, Gallois P (2004) Ultraviolet-C overexposure induces programmed cell death in Arabidopsis, which is mediated by caspase-like activities and which can be suppressed by caspase inhibitors, p35 and Defender against Apoptotic Death. J Biol Chem 279: 779–787. 14573611 10.1074/jbc.M304468200

[68] Hatsugai N, Iwasaki S, Tamura K, Kondo M, Fuji K, et al (2009) A novel membrane fusion-mediated plant immunity against bacterial pathogens. Gene Dev 23: 2496–2506. 19833761 10.1101/gad.1825209 PMC2779742

[69] Takayama S, Sato T, Krajewski S, Kochel K, Irie S, et al (1995) Cloning and functional-anlaysis of BAG-1 - A novel BCL-2-binding protein with anti-cell death activity. Cell 80: 279–284. 7834747 10.1016/0092-8674(95)90410-7

[70] Doukhanina EV, Chen S, van der Zalm E, Godzik A, Reed J, et al (2006) Identification and functional characterization of the BAG protein family in Arabidopsis thaliana. J Biol Chem 281: 18793–18801. 16636050 10.1074/jbc.M511794200

[71] Kang CH, Jung WY, Kang YH, Kim JY, Kim DG, et al (2006) AtBAG6, a novel calmodulin-binding protein, induces programmed cell death in yeast and plants. Cell Death Differ 13: 84–95. 16003391 10.1038/sj.cdd.4401712

[72] Sun L, Lu SJ, Zhang SS, Zhou SF, Sun L, et al (2013) The lumen-facing domain is important for the biological function and organelle-to-organelle movement of bZIP28 during ER Stress in Arabidopsis. Mol Plant 6: 1605–1615. 23558471 10.1093/mp/sst059

[73] Srivastava R, Deng Y, Shah S, Rao AG, Howell SH (2013) BINDING PROTEIN is a master regulator of the endoplasmic reticulum stress sensor/transducer bZIP28 in Arabidopsis. Plant Cell 25: 1416–1429. 23624714 10.1105/tpc.113.110684 PMC3663277

[74] Gardner BM, Walter P (2011) Unfolded proteins are Ire1-activating ligands that directly induce the unfolded protein response. Science 333: 1891–1894. 21852455 10.1126/science.1209126 PMC3202989

[75] Kim YS, Kim SG, Park JE, Park HY, Lim MH, et al (2006) A membrane-bound NAC transcription factor regulates cell division in Arabidopsis. Plant Cell 18: 3132–3144. 17098812 10.1105/tpc.106.043018 PMC1693948

[76] Kim SG, Lee AK, Yoon HK, Park CM (2008) A membrane-bound NAC transcription factor NTL8 regulates gibberellic acid-mediated salt signaling in Arabidopsis seed germination. Plant J 55: 77–88. 18363782 10.1111/j.1365-313X.2008.03493.x

[77] Ng S, Ivanova A, Duncan O, Law SR, Van Aken O, et al (2013) A membrane-bound NAC transcription factor, ANAC017, mediates mitochondrial retrograde signaling in Arabidopsis. Plant Cell 25: 3450–3471. 24045017 10.1105/tpc.113.113985 PMC3809543

[78] De Clercq I, Vermeirssen V, Van Aken O, Vandepoele K, Murcha MW, et al (2013) The membrane-bound NAC transcription factor ANAC013 functions in mitochondrial retrograde regulation of the oxidative stress response in Arabidopsis. Plant Cell 25: 3472–3490. 24045019 10.1105/tpc.113.117168 PMC3809544

[79] Li P, Wind JJ, Shi X, Zhang H, Hanson J, et al (2011) Fructose sensitivity is suppressed in Arabidopsis by the transcription factor ANAC089 lacking the membrane-bound domain. Proc Natl Acad Sci USA 108: 3436–3441. 21300879 10.1073/pnas.1018665108 PMC3044370

[80] Zhang C, Chen X, Zhu RM, Zhang Y, Yu T, et al (2012) Endoplasmic reticulum stress is involved in hepatic SREBP-1c activation and lipid accumulation in fructose-fed mice. Toxi Lett 212: 229–240. 10.1016/j.toxlet.2012.06.002 22698815

[81] Klein P, Seidel T, Stocker B, Dietz KJ (2012) The membrane-tethered transcription factor ANAC089 serves as redox-dependent suppressor of stromal ascorbate peroxidase gene expression. Front Plant Sci 3: 247–247. 23162559 10.3389/fpls.2012.00247 PMC3493970

[82] Lin JH, Li H, Yasumura D, Cohen HR, Zhang C, et al (2007) IRE1 signaling affects cell fate during the unfolded protein response. Science 318: 944–949. 17991856 10.1126/science.1146361 PMC3670588

[83] Howell SH (2013) Endoplasmic reticulum stress responses in plants. Annual Rev Plant Biol 64: 477–499. 23330794 10.1146/annurev-arplant-050312-120053

[84] Qiang X, Zechmann B, Reitz MU, Kogel K-H, Schaefer P (2012) The mutualistic fungus piriformospora indica colonizes Arabidopsis roots by inducing an endoplasmic reticulum stress-triggered caspase-dependent cell death. Plant Cell 24: 794–809. 22337916 10.1105/tpc.111.093260 PMC3315247

[85] Liu Y, Burgos JS, Deng Y, Srivastava R, Howell SH, et al (2012) Degradation of the endoplasmic reticulum by autophagy during endoplasmic reticulum stress in Arabidopsis. Plant Cell 24: 4635–4651. 23175745 10.1105/tpc.112.101535 PMC3531857

[86] Sun L, Yang ZT, Song ZT, Wang MJ, Lu SJ, et al (2013) The plant-specific transcription factor NAC103 is induced by bZIP60 through a new cis-regulatory element to modulate the unfolded protein response in Arabidopsis. Plant J 76: 274–286. 23869562 10.1111/tpj.12287

[87] Liu JX, Srivastava R, Howell SH (2008) Stress-induced expression of an activated form of AtbZIP17 provides protection from salt stress in Arabidopsis. Plant Cell Environ 31: 1735–1743. 18721266 10.1111/j.1365-3040.2008.01873.x

[88] Iwata Y, Fedoroff NV, Koizumi N (2008) Arabidopsis bZIP60 is a proteolysis-activated transcription factor involved in the endoplasmic reticulum stress response. Plant Cell 20: 3107–3121. 19017746 10.1105/tpc.108.061002 PMC2613661

[89] Hellens RP, Allan AC, Friel EN, Bolitho K, Grafton K, et al (2005) Transient expression vectors for functional genomics, quantification of promoter activity and RNA silencing in plants. Plant Methods 1: 13. 16359558 10.1186/1746-4811-1-13 PMC1334188

