# Supplementary figures and images for: The Membrane-Associated Transcription Factor NAC089 Controls ER-Stress-Induced Programmed Cell Death in Plants
Source: PLoS Genet. 2014 Mar 27;10(3):e1004243. doi: 10.1371/journal.pgen.1004243 (PMC3967986; doi:10.1371/journal.pgen.1004243)

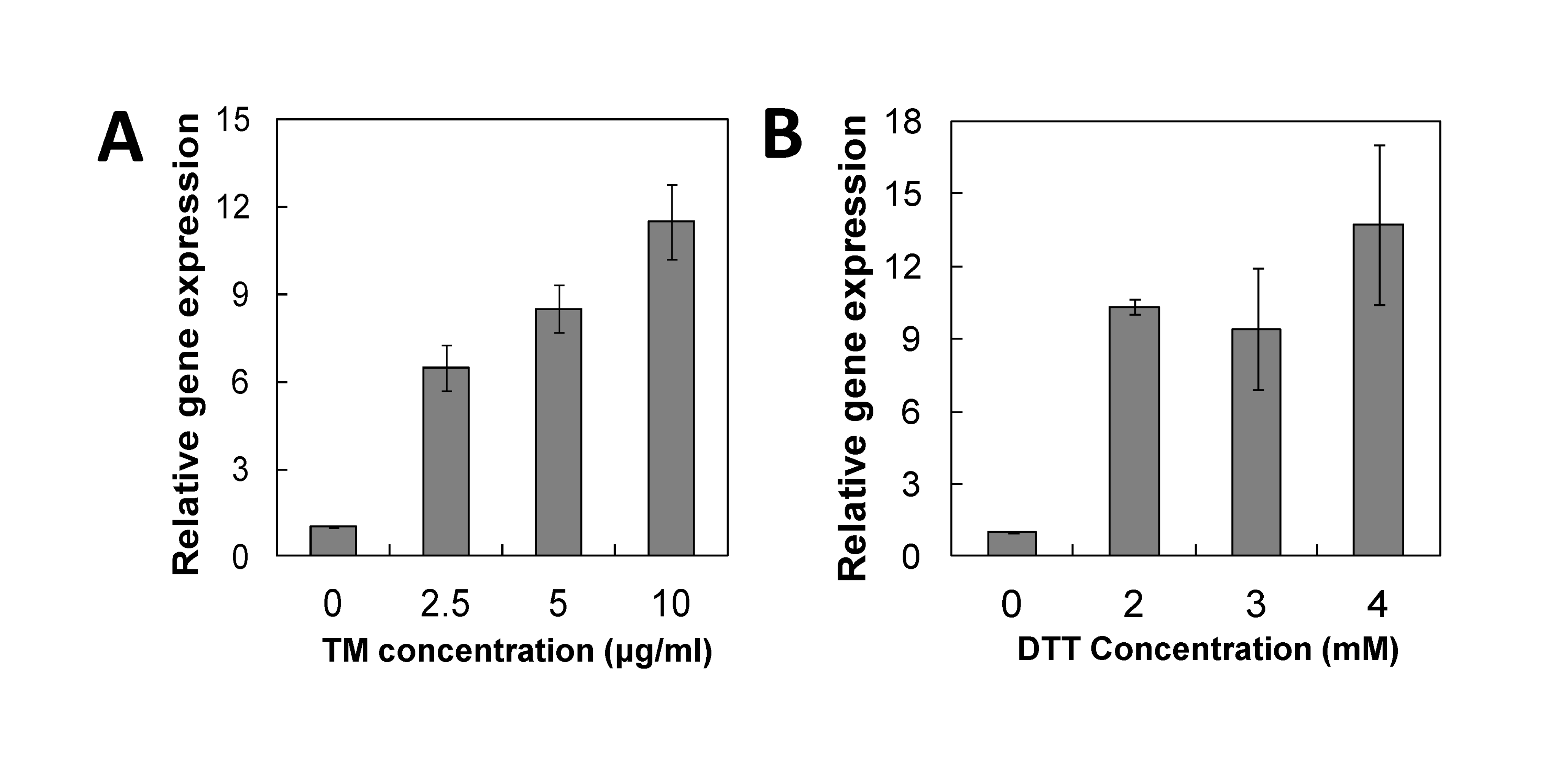

Supplement: Figure S1 — NAC089 is up-regulated by ER stress. (A–B) Up-regulation of NAC089 by tunicamycin (TM, A) and dithiothreitol (DTT, B) was examined in the dose-response experiments. The expression of NAC089 is normalized to the expression of the internal control actin. The wild-type Arabidopsis seedlings were stressed for 4 hr. Bars depict SE (n = 3). (TIF) [file pgen.1004243.s002.tif]

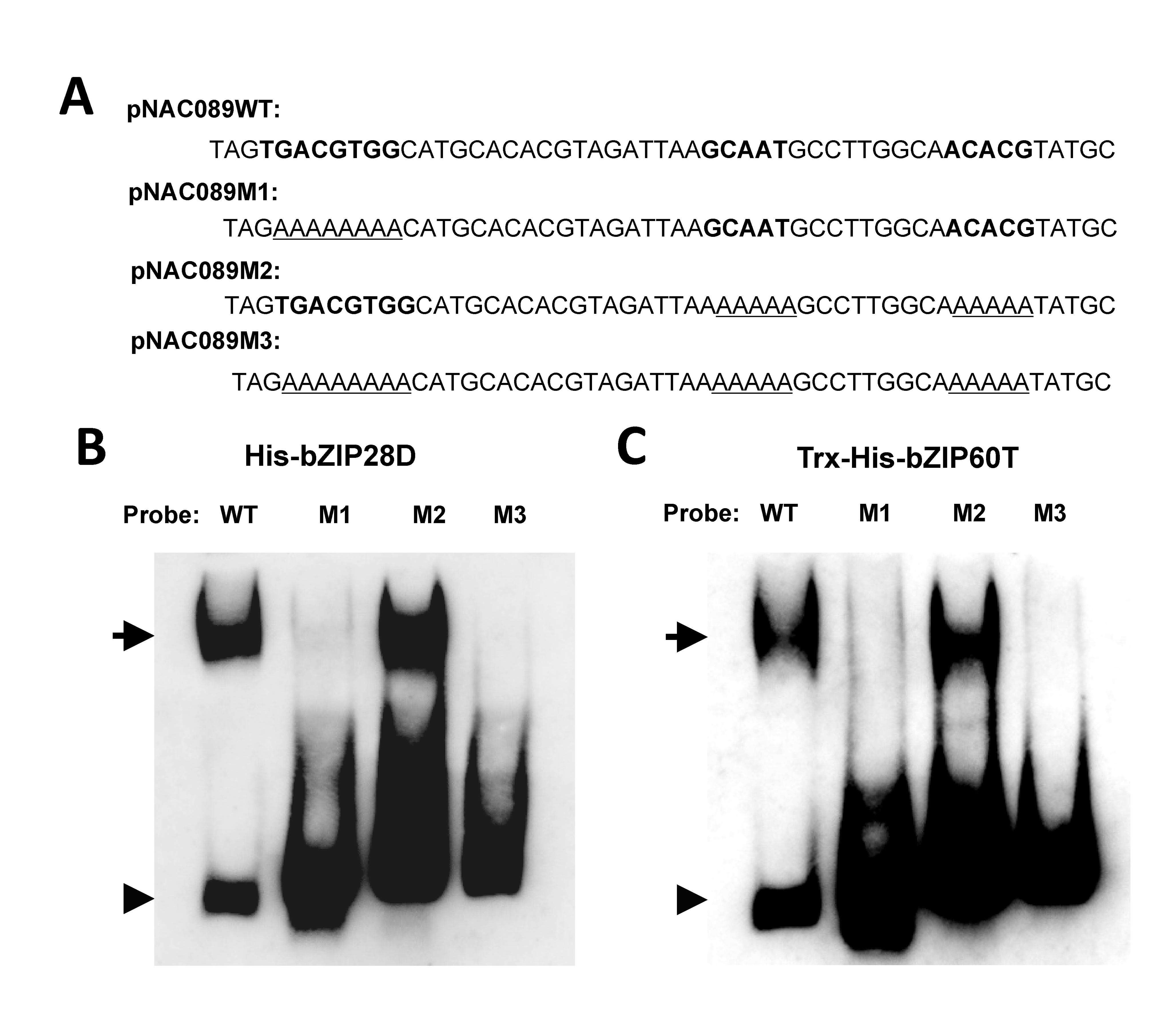

Supplement: Figure S2 — bZIP28 and bZIP60 bind to UPRE but not to ERSE-I-like cis-element. (A) DNA sequences of biotin-labeled probes. The predicted ER stress responsive cis-elements UPRE and ERSE-I-Like were highlighted in bold. The mutated sites were underlined. (B–C) EMSA for protein and DNA interactions. Either the purified His-bZIP28D (B) or Trx-His-bZIP60T (C) was incubated with various biotin-labeled DNA. Arrows and arrow heads point to the positions of shifted bands and free probes, respectively. (TIF) [file pgen.1004243.s003.tif]

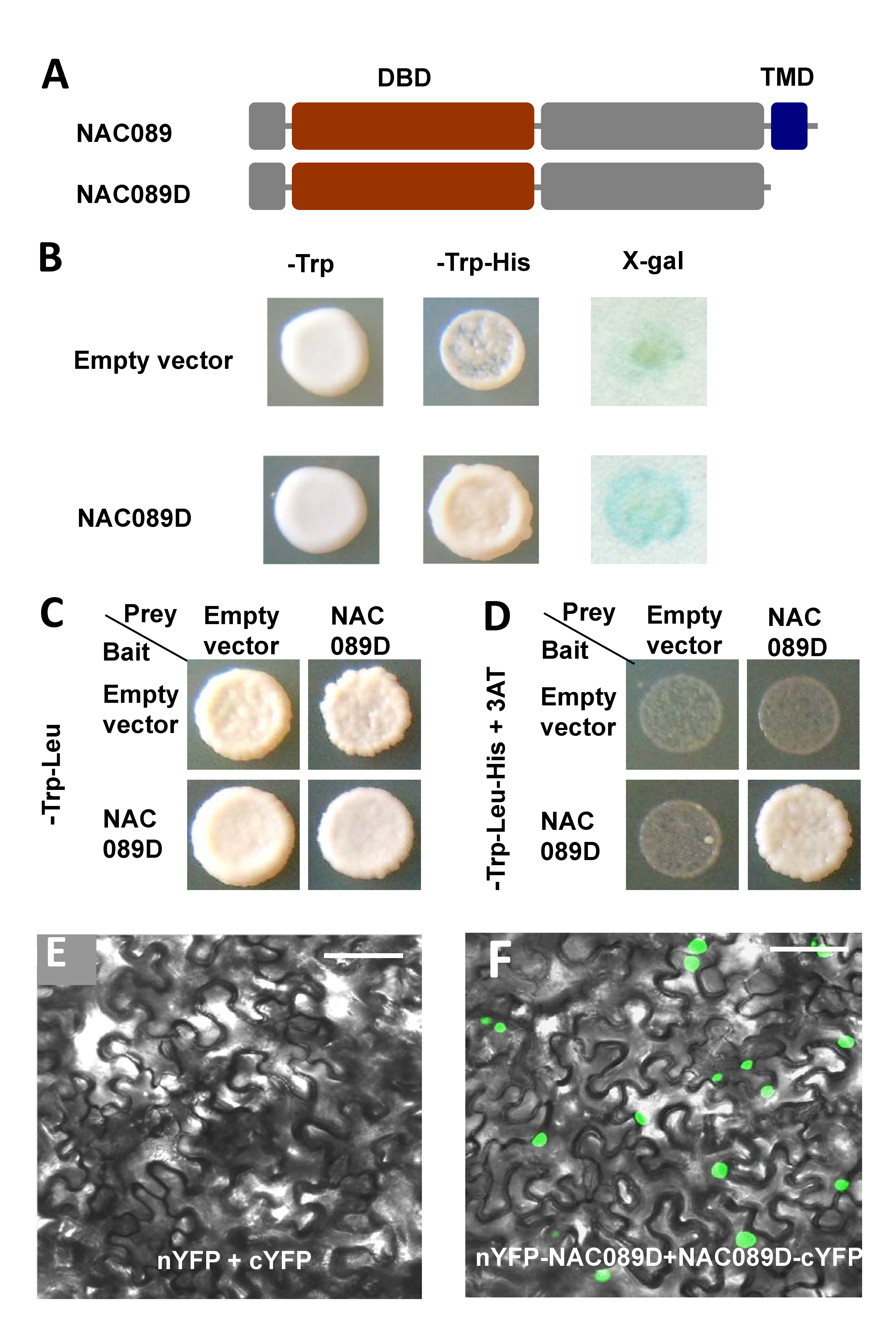

Supplement: Figure S3 — NAC089 has transcriptional activation activity and forms homodimmers. (A) Schematic structure of NAC089 protein. DBD: DNA binding domain; TMD: transmembrane domain. (B) Transcriptional activation activity of NAC089. Segment of NAC089 lacking the TMD (NAC089D) was fused to yeast GAL4 DNA binding domain and the activation of HIS and LacZ reporters were evaluated. (C–F) Evaluation of dimmer formation in yeast 2-hybrid assays (C–D) and BiFC assays (E–F). Bar = 50 µm. (TIF) [file pgen.1004243.s004.tif]

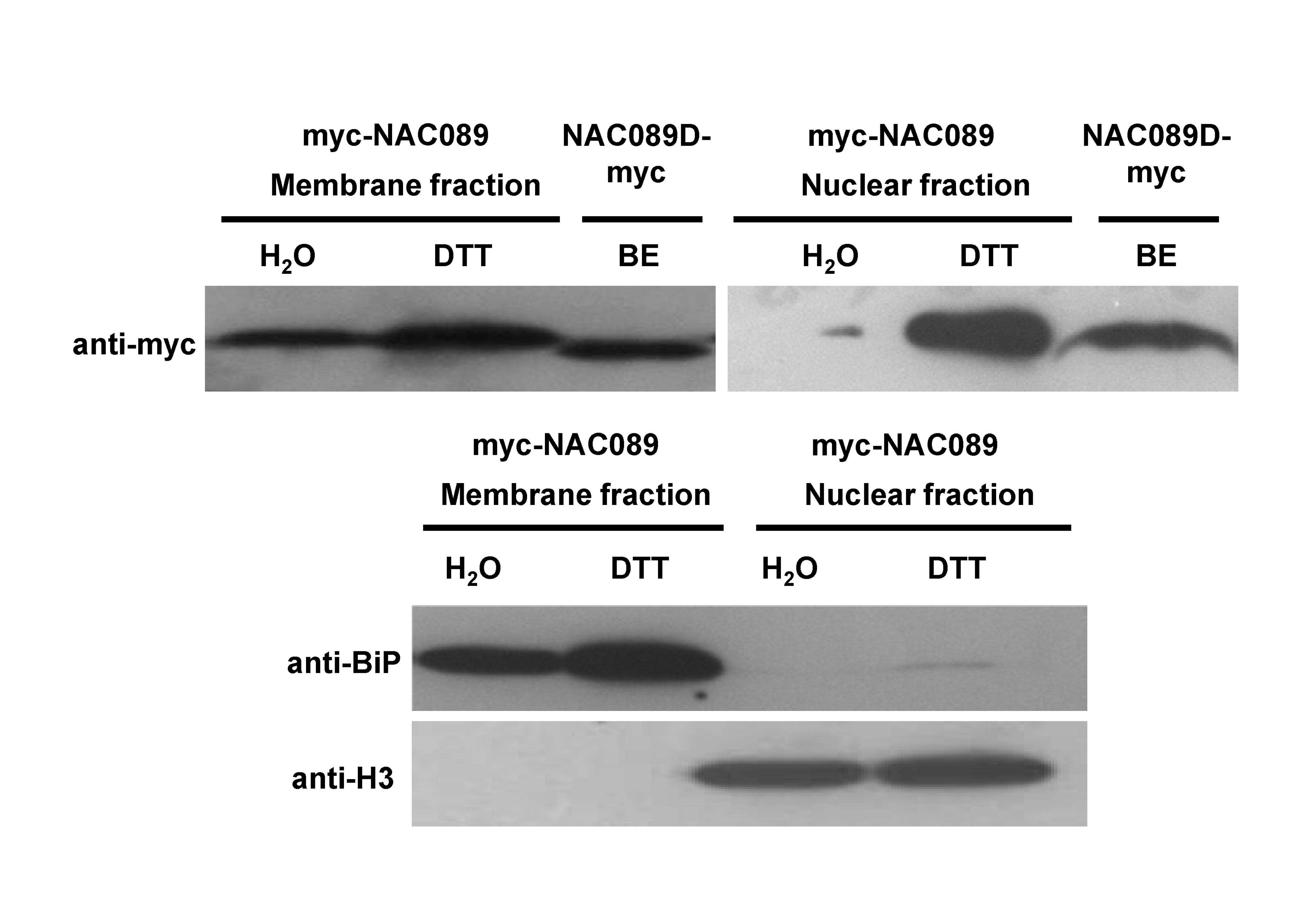

Supplement: Figure S4 — NAC089 is enriched in the membrane fraction or nuclear fraction depending on different conditions. Plant seedlings were treated with H2O (control), TM or DTT for 6 hr and the used for protein fractionation studies. Anti-Histone and anti-BiP antibodies were employed to detect the nuclear protein marker Histone H3 and ER protein marker BiP, respectively. The beta-estradiol (BE) induced truncated form NAC089D-MYC was used as the migration marker. (TIF) [file pgen.1004243.s005.tif]

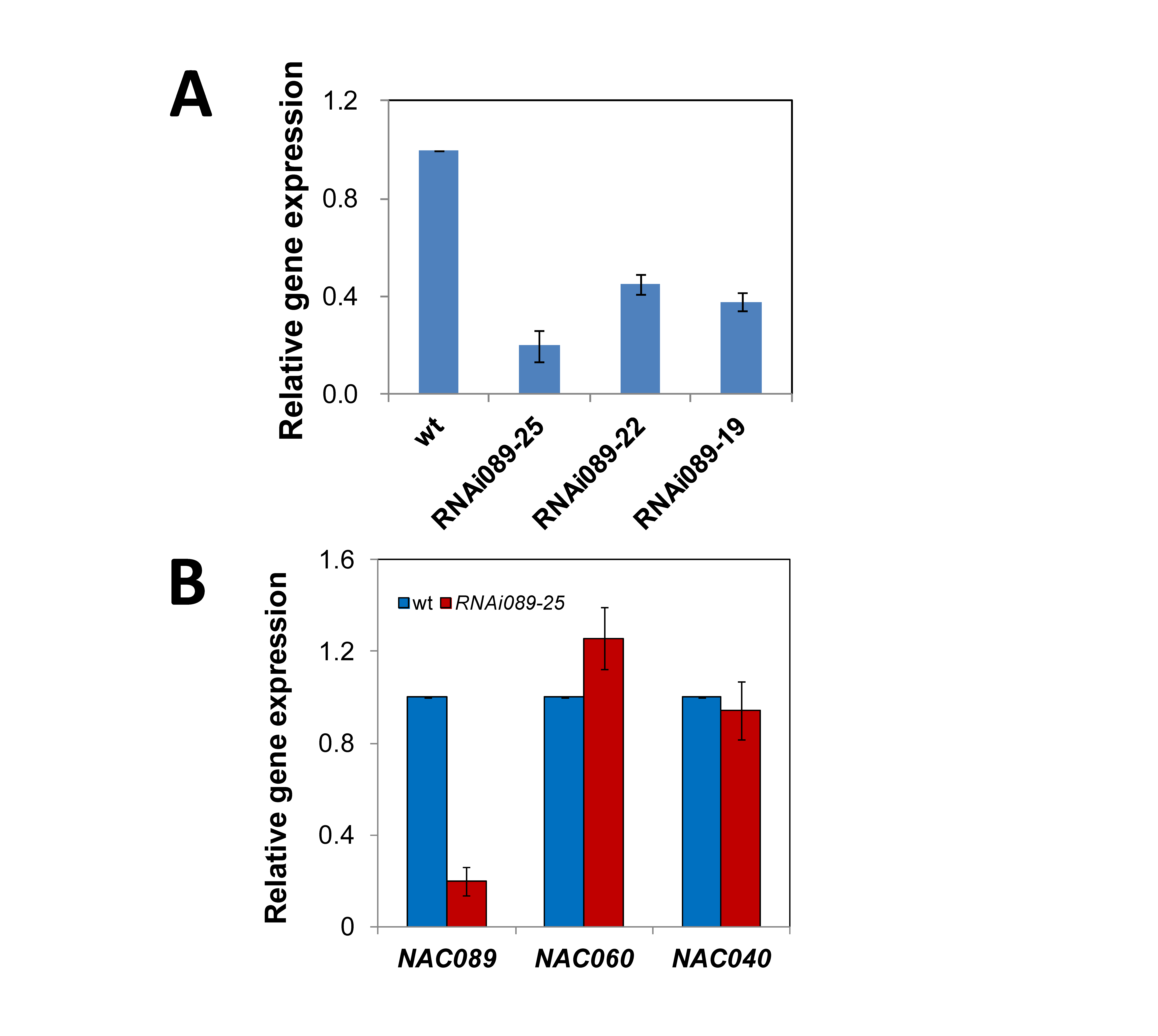

Supplement: Figure S5 — NAC089 is specifically knocked-down in the RNAi plants. (A–B) The expression of NAC089 (A) and its close-related homologs NAC060 and NAC040 (B) in the NAC089 knock-down plants. The relative gene expression in the transgenic plants is the value normalized to the expression in the wild-type control (wt), both of which are normalized to the internal control actin. Bars depict SE (n = 3). (TIF) [file pgen.1004243.s006.tif]

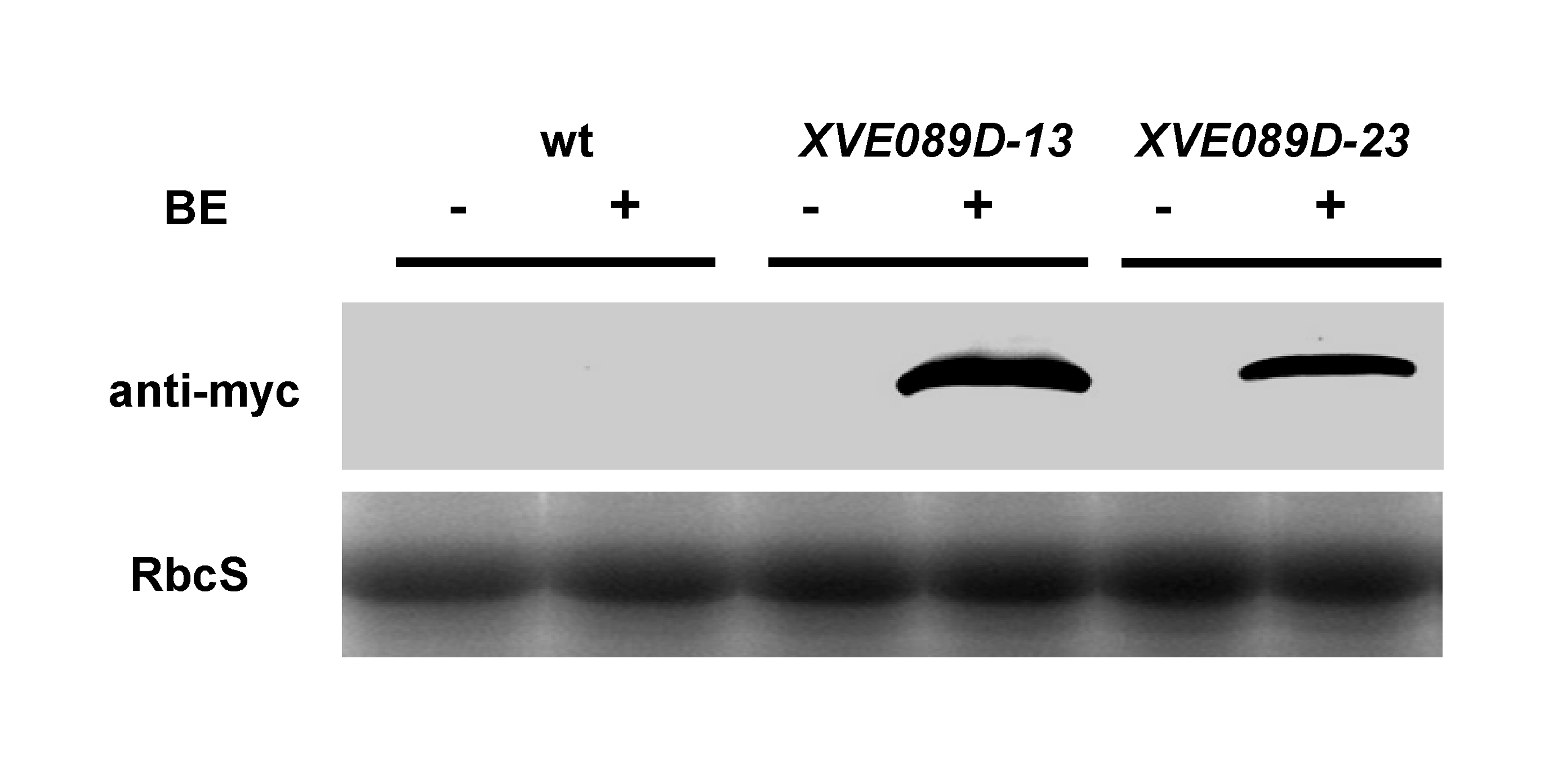

Supplement: Figure S7 — NAC089D-MYC is expressed in the overexpression plants. Validation of transgenic expression in two lines of NAC089D-MYC overexpression plants by western blotting. Coomassie blue staining of RbcS serves as a loading control for western blotting. Plant seedlings were treated with beta-estradiol (BE) for 16 hr. (TIF) [file pgen.1004243.s008.tif]

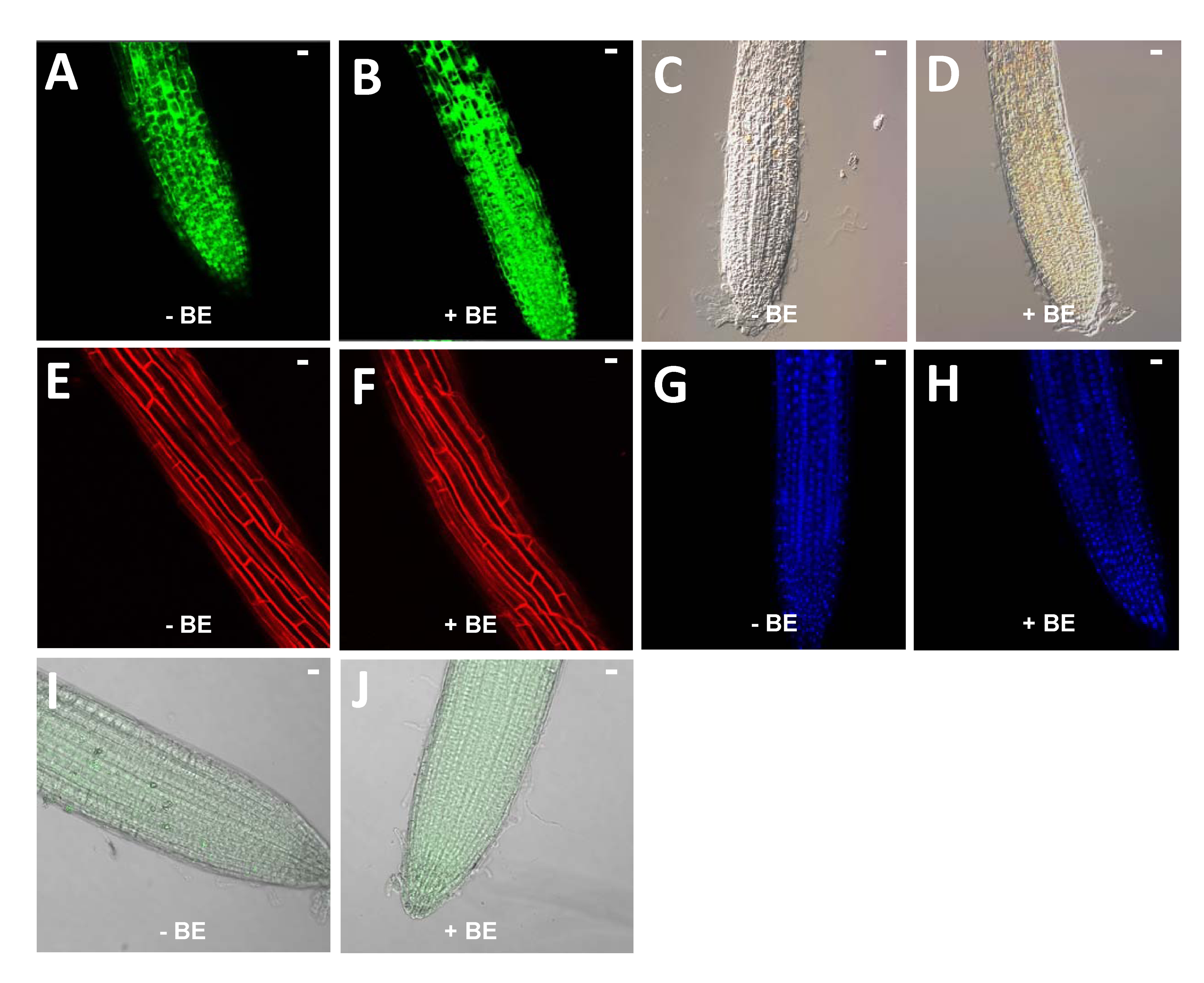

Supplement: Figure S8 — Beta estradiol has little effect on histochemical staining in the wild-type control plants. (A–D) Esterase activities (A–B) and H2O2 accumulation (C–D) in the wild-type control roots as revealed by FDA and DAB staining without (A, C) or with (B, D) beta-estradiol (BE) treatment for 3 days. (E–H) Membrane rigidity and nucleus diffusion in the wild-type control roots as reflected by PI (E–F) and DAPI (G–H) staining without (E, G) or with (F, H) BE treatment for 5 days. (I–J) DNA breakage in the wild-type control roots as detected by TUNEL assay without (I) or with (J) BE treatment for 5 days. Bar = 10 µm. (TIF) [file pgen.1004243.s009.tif]

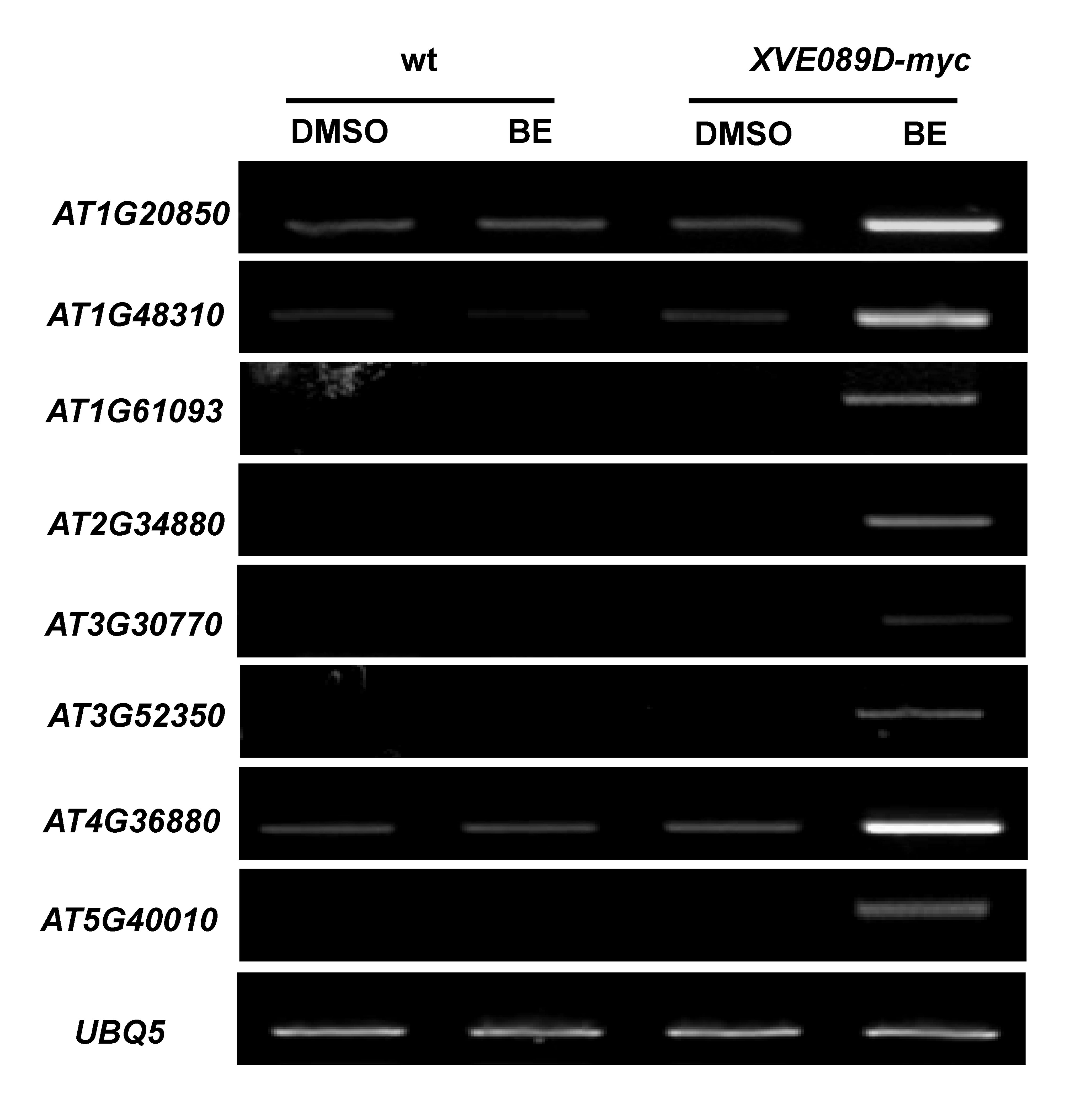

Supplement: Figure S9 — Beta estradiol does not induce NAC089 downstream genes in the wild-type control plants. Wild-type control (wt) and XVE089D-MYC plants were treated with DMSO (control) or beta-estradiol (BE) for 16 hr and gene expressions were checked with RT-PCR. UBQ5 was used as an internal control (TIF) [file pgen.1004243.s010.tif]

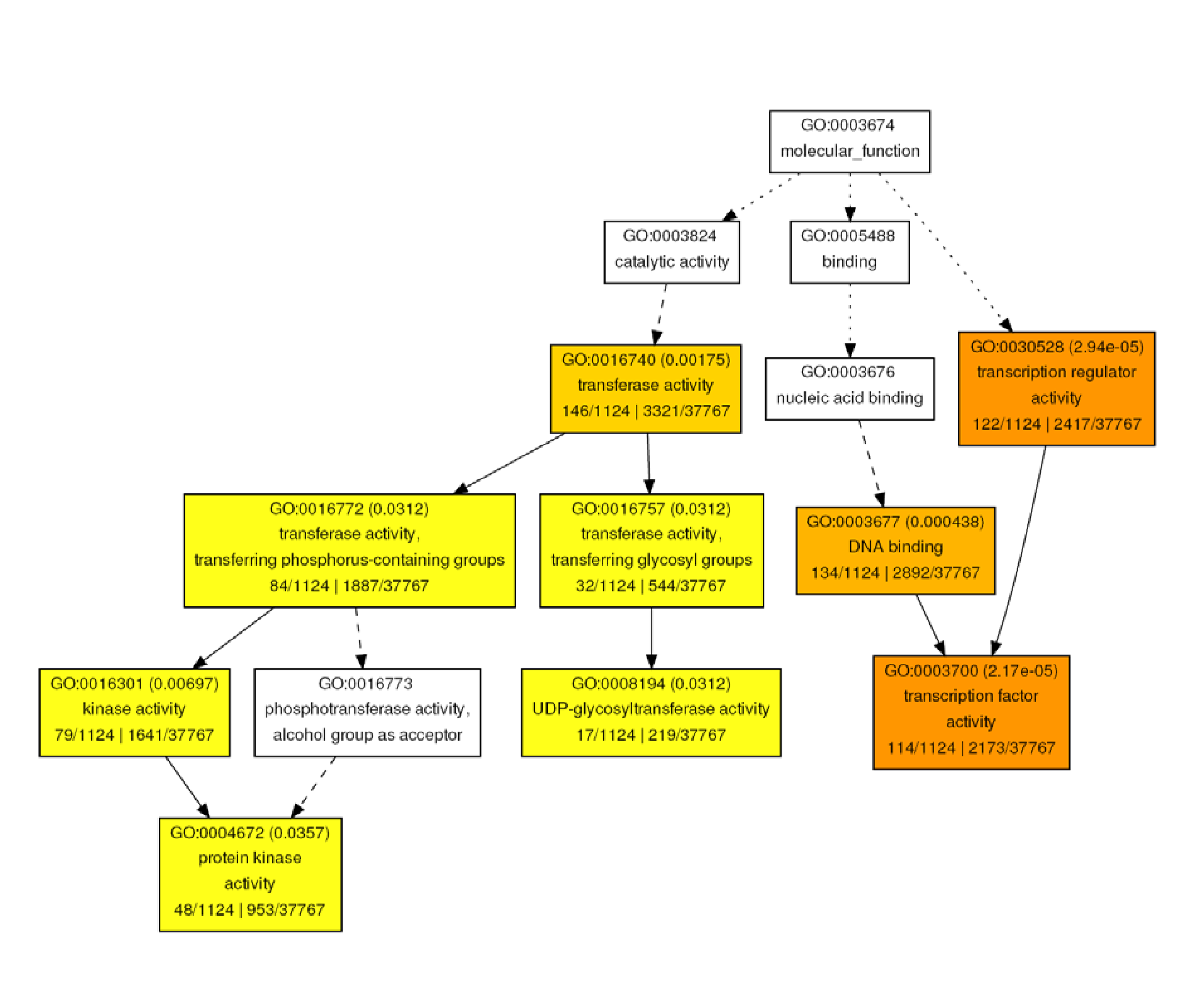

Supplement: Figure S10 — Transcription factor activity is enriched in the NAC089 downstream genes. Gene Ontology (GO) analysis was performed with NAC089D-MYC induced genes in agriGO. (TIF) [file pgen.1004243.s011.tif]

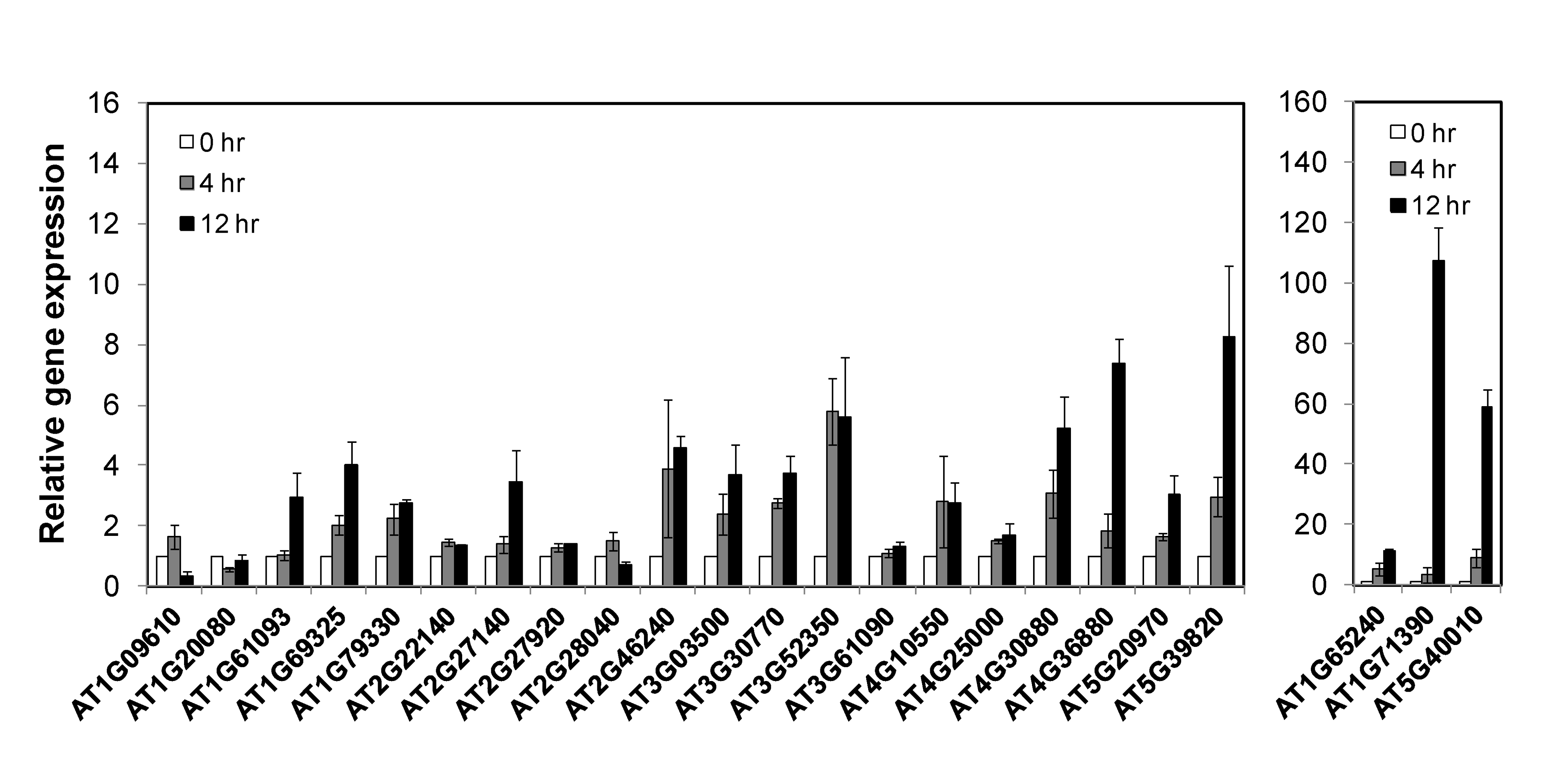

Supplement: Figure S11 — Some of the NAC089D-MYC-regulated genes are up-regulated by ER stress. Totally 23 genes were selected from the microarray experiment and their expressions were examined with qRT-PCR. The wild-type plants were treated with tunicamycin for 4 and 12 hr and the expression of NAC089 target genes was also quantified with qRT-PCR. The relative gene expression is the value in the treated sample normalized to the untreated control, both of which are normalized to the expression of actin. Bars depict SE (n = 3). (TIF) [file pgen.1004243.s012.tif]

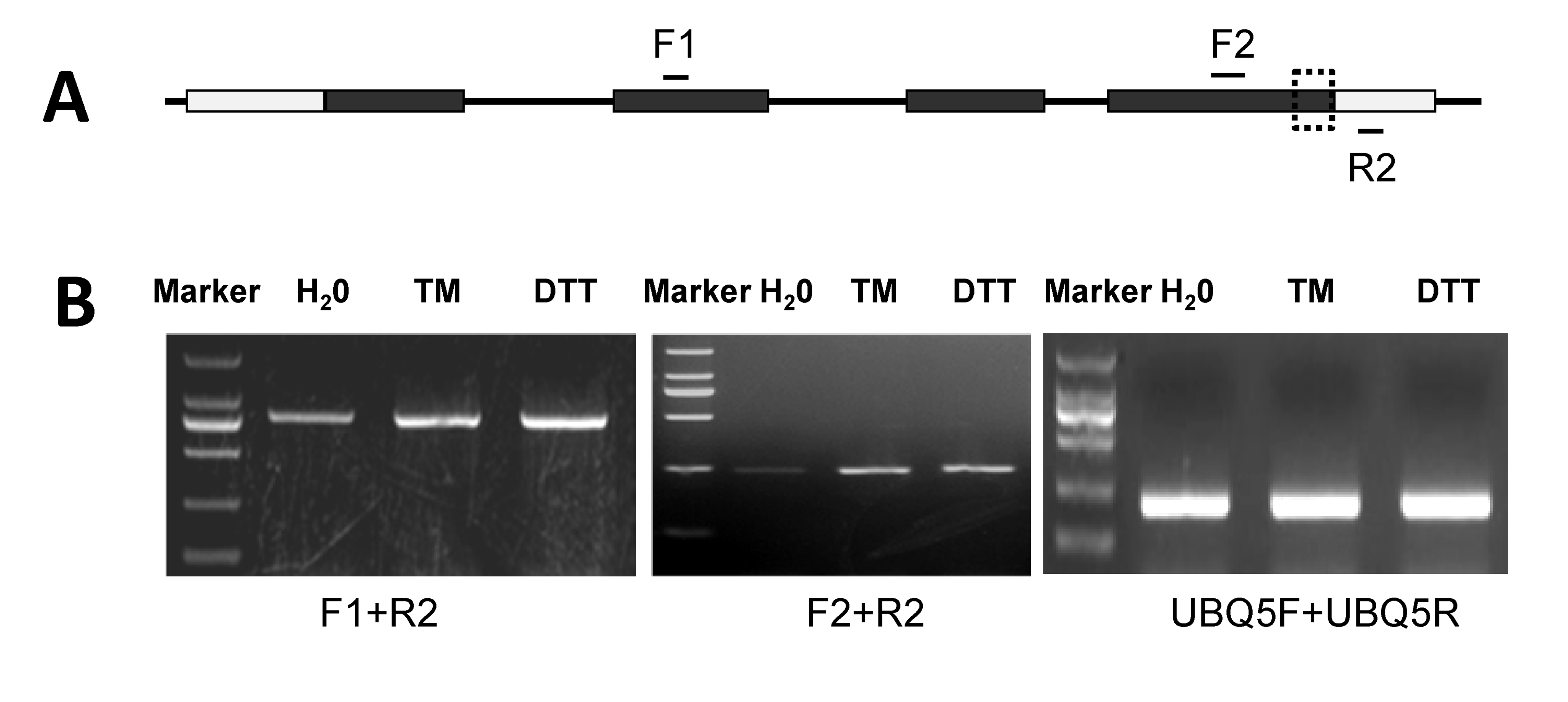

Supplement: Figure S12 — NAC089 is not alternatively spliced under ER stress condition. (A) Gene model of NAC089. White rectangles represent UTRs and black rectangles denote exons. The region encoding the transmembrane domain of NAC089 is boxed. (B) Detection of NAC089 transcript with RT-PCR. The wild-type plants were treated with H20 (control), 5 µg/ml tunicamycin (TM) or 2 mM DTT for 4 hr and the expression of NAC089 was examined by RT-PCR with different primer pairs. UBQ5 was used as a loading control. (TIF) [file pgen.1004243.s013.tif]
