# Supplementary material for: The Membrane-Associated Transcription Factor NAC089 Controls ER-Stress-Induced Programmed Cell Death in Plants
Source: PLoS Genet. 2014 Mar 27;10(3):e1004243. doi: 10.1371/journal.pgen.1004243 (PMC3967986; doi:10.1371/journal.pgen.1004243)
Supplement: Table S1 — Primers used in the study. (DOC) [file pgen.1004243.s014.doc]

**Table S1.** Primers used in the study

| **Name** | **Purpose** | **Forward Primer** | **Reverse Primer** |
| --- | --- | --- | --- |
| NAC089 | CDS cloning | TTGGCGCGCCATGGACACGAAGGCGGTTGG | GGACTAGTTATTCTAGATAAAACAACATT |
| GFP | GFP tagging | tgcctcgagatgagtaaaggagaagaacttttca | gaaggcgcgccctttgtatagttcatccatgccatg |
| MYC | myc tagging | gaaggcgcgccatggaacagaaattaatctctgaag | gaaggcgcgcccaaggtcttcttcagagataagttt |
| RNAi089 | RNAi silencing | ccgCTCGAGGGATCCGAGTGGTACAAGTCAGAAGGC | ggGGTACCATCGATTTGCTATCAGAGCCACTACACC |
| EAR | EAR tagging | GATCCCAAGATCTGGATCTAGAACTCCGTTTGGGTTTCGCTTAACTGCA | GTTAAGCGAAACCCAAACGGAGTTCTAGATCCAGATCTTGG |
| PRO089 | Promoter cloning | GGGAATTCCTTGATCTATATCCTTGGATGG | CGGAGCTCCTTCACAGGTGCACACATATGG |
| EAR089 | NAC089-EAR | CCGAATTCATGGACACGAAGGCGGTTGG | CCGGATCCTTTGATCGAGTATAAACCCA |
| XVE089 | Overexpression | ttggcgcgccATGGACACGAAGGCGGTTGG | ggactagtTTTGATCGAGTATAAACCCACG |
| Y2H089BD | Y2H | CCGAATTCATGGACACGAAGGCGGTTGG | CCGGATCCTTTGATCGAGTATAAACCCA |
| Y2H089AD | Y2H | CCGAATTCATGGACACGAAGGCGGTTGG | CCGGATCCTTTGATCGAGTATAAACCCA |
| BiFC089N | BiFC | CGGGATCCATGGACACGAAGGCGGTTGG | GCTCTAGATTTGATCGAGTATAAACCCA |
| BiFC089C | BiFC | CGGGATCCATGGACACGAAGGCGGTTGG | GCTCTAGATTATTTGATCGAGTATAAACCCA |
| pNAC089 | Dual luciferase | AGCTTTAGTGACGTGGCATGCACACGTAGATTAAGCAATGCCTTGGCAACACGTATGCA | CTAGTGCATACGTGTTGCCAAGGCATTGCTTAATCTACGTGTGCATGCCACGTCACTAA |
| Pet28-60 | Expression | CGGGATCCATGACGGAATCAACATCCGTGG | GCAAGCTTGCGGCCGCTCACTTCTTGAGCTTACTT |
| Pet28-28 | Expression | GAAGGCGCGCCATGGCGGAGGAATTTGGAAG | ATAGTCGACTCAAGCAGACTCCTGCTTCGACA |
| NAC089S1 | Splicing assay | ACTGGGAAAGAGCGTGATGT | ATAATCCTTGTCAATGTCCG |
| NAC089S2 | Splicing assay | ATCACTGTTAGAGGAAAGAC | ATAATCCTTGTCAATGTCCG |
| UBQ5 | RT-PCR | ttgaagacggccgtaccctc | cgctgaacctttcaagatccatcg |
| AT5G22290 | RT-PCR | GCCAACACTAGAATCAGCATCA | TTGCTATCAGAGCCACTACACC |
| AT1G20850 | RT-PCR | CATTGATGCATCTGGTAGAG | CCAGTGTTCCTCTTCAGCCT |
| AT1G48310 | RT-PCR | GAAGCTCCCAATCTCCATCT | TAACCAGCTTGAGCATCCTA |
| AT1G61093 | RT-PCR | CGGTTATTCAGAAACAGCTC | CTTACACTCTTGGGTACGTA |
| AT2G34880 | RT-PCR | TCTAGACACTTTCCGCACAA | ACACAGAACAAAGCCAATCG |
| AT3G30770 | RT-PCR | ATGAGTGGTGAAAGTCTTGC | TCACTCCTAGACTACCCTGT |
| AT3G52350 | RT-PCR | TCCCAGGCTACAATTTCTCA | CTTCCAGCCATGCTTCTTTA |
| AT4G36880 | RT-PCR | ATGGTGGCTTAAACACTGAGA | CGGCTTCAATAGCTACACTA |
| AT5G40010 | RT-PCR | GACTAATACGGGCTCTGCTC | CATAGACATCGCTTCGCTTG |
| ACTIN | qRT-PCR | ggtaacattgtgctcagtggtgg | aacgaccttaatcttcatgctgc |
| AT1G09610 | qRT-PCR | TCTTGCCACCTTAATTCTCA | TCTACTGCTTACCGCTATCT |
| AT1G20080 | qRT-PCR | TCTACTGGTGGCTTGCTTGT | GGCTCATCTAATGGGAACTG |
| AT1G61093 | qRT-PCR | CGGTTATTCAGAAACAGCTC | CTTACACTCTTGGGTACGTA |
| AT1G65240 | qRT-PCR | TGTACTGCTTTGGTTGGCAA | CCGAGTAAACACCTCCTGAT |
| AT1G69325 | qRT-PCR | TGGGAAGATAAGAAGAGGAA | CAGAGGCATGTAGAGGGTTT |
| AT1G71390 | qRT-PCR | GGACGTAAGTGGCAACCAGT | CATGCTGGGATGATACAACG |
| AT1G79330 | qRT-PCR | CTAACAAAGCTGCAAGAAGG | CTAACAAAGCTGCAAGAAGG |
| AT2G22140 | qRT-PCR | CCTCTTGCCAATTCAGGAAG | CACTGCTAAAGCGTATCGTG |
| AT2G27140 | qRT-PCR | GTCCAGCAGGAGCCAACAAA | TAGGAAGCCTCACAACGAGA |
|  |  |  |  |
| **Table S1.** (continued). | | | |
| **Name** | **Purpose** | **Forward Primer** | **Reverse Primer** |
| AT2G27300 | qRT-PCR | TTGCCAGAGGAATCGAAACT | CCTCCTTCTCCAAACCACCT |
| AT2G27920 | qRT-PCR | CGTCGATGAGCTTCTTGCCA | AGCCTCTTGTTGCTCTGTCA |
| AT2G28040 | qRT-PCR | TGCAACCTAGTAAGAAAGGC | AACTCCTCCTGTATTCGATG |
| AT2G46240 | qRT-PCR | GTGAAGGGAATATGACTGCA | CTTCTGTCTCTAGAGGCTGT |
| AT3G03500 | qRT-PCR | GTTCAGCTGAAGTTGTTCCT | GATCCGAAGAACTCTCATCT |
| AT3G30770 | qRT-PCR | ATGAGTGGTGAAAGTCTTGC | TCACTCCTAGACTACCCTGT |
| AT3G44290 | qRT-PCR | CTTCACGAGTCTTGTTCCGA | GATATAAGTTCGATGTCGGT |
| AT3G52350 | qRT-PCR | TCCCAGGCTACAATTTCTCA | CTTCCAGCCATGCTTCTTTA |
| AT3G61090 | qRT-PCR | TCAACATTCTCGTAGCACAG | CTAAGGTCTGGTTCTTCTTG |
| AT4G10550 | qRT-PCR | TGGACCTCTCAACTCAGTCT | CTTCCAAAGTAGTAACCTGT |
| AT4G25000 | qRT-PCR | AACGCTGGAATCACTCATCT | TCTGAACCGTATTTGGAGCT |
| AT4G30880 | qRT-PCR | GGATAAGGTTGTTCATGTCA | TCAAACCCTTAGCTCTCAAG |
| AT4G36880 | qRT-PCR | ATGGTGGCTTAAACACTGAGA | CGGCTTCAATAGCTACACTA |
| AT5G20970 | qRT-PCR | CCGAGGTTCTTGTCGCTGAT | AGTTGGGCGTTCTCCTGTTA |
| AT5G22290 | qRT-PCR | ACTGGGAAAGAGCGTGATGT | TGGTGCCTTCTGACTTGTAC |
| AT5G39820 | qRT-PCR | TCCAACGATTCCTGACAACA | TCGTTGACTGGAGAAGGAAT |
| AT5G40010 | qRT-PCR | GACTAATACGGGCTCTGCTC | CATAGACATCGCTTCGCTTG |
| AT1G09090 | qRT-PCR | AAGAGCGGCATTGACATCGT | TGGAACTCAAACTTGGTCGT |
| At1g09080 | qRT-PCR | CACGGTTCCAGCGTATTTCAAT | ATAAGCTATGGCAGCACCCGTT |
| At1g21750 | qRT-PCR | CTCGTGAAGCTGAGGGTATTG | TGTGCGAAATCTAACTCAGAG |
| AT1G56340 | qRT-PCR | AGACCTTAGTCTTCCAATTCTC | CCATTGTAAGTAAGGATAGCATG |
| AT1G77510 | qRT-PCR | TAGTGTACGAAGGAGACAGG | GAAGAAGAGAAACGCTCCTA |
| AT2G38470 | qRT-PCR | CGATTCGTTTATGCCCTGAA | CTGACTTACTATCCTGCAGG |
| AT3G08970 | qRT-PCR | TCAGCTAAA GGTTTACGCGG | CCATCAGTTCTCTCCAAGCT |
| AT5G28540 | qRT-PCR | TCACTTGGGAGGTGAGGACTTT | CTCACATTCCCTTCGGAGCTTA |
| AT5G47120 | qRT-PCR | CTGACTTTGTAGCTGTGTTT | CTGAAGATGGTACTTGTGCA |
| At5g61790 | qRT-PCR | ATGAGACAACGGCAACTATTTTCC | CCATAATCCTCATGTCCTTCACT |
| AT1G65240 | ChIP-q-PCR | GTCAATTGTTCCCAAAATGG | CACAGAACGCGTTTTCAAAG |
| AT1G69325 | ChIP-q-PCR | TCCTCCAACCGGTAAGATTA | AGATAGATTCCTTGTCTCCT |
| AT1G71390 | ChIP-q-PCR | CATGAGACAGTAAACGCGTA | GAGTTATATGTGCTTCACTG |
| AT1G79330 | ChIP-q-PCR | GACAAGCCATAGACGTTTTC | AGCCTTAATAAGGTTGCCAG |
| AT2G27140 | ChIP-q-PCR | AATGCTTGGGAGAGACGTGA | TTGTGGAGATGGCTTCATGT |
| AT2G46240 | ChIP-q-PCR | CTCACTGCTTCAAAGCTTTG | GCTGTATATTGAGCGTTAGT |
| AT3G03500 | ChIP-q-PCR | AAGCCATATGGGAGGAGCTT | TTGTTGGCATTGAAGTTTCG |
| AT3G30770 | ChIP-q-PCR | CACCACAGCTCCTCTGTTGA | AGCCCAATAGAAGCCCGATA |
| AT3G52350 | ChIP-q-PCR | GTGGGTACGGGGCATATAAG | GAACTTCTTCCGTTTAGGGT |
| AT4G30880 | ChIP-q-PCR | GGCTGCCATGTAGTTGTAGT | GCTTTGGAGGTTTTAAGGGT |
| AT4G36880 | ChIP-q-PCR | AAAATCCATCGAACCGTCCA | GGAGGATGAGAAAACTGTGG |
| AT5G39820 | ChIP-q-PCR | ATTGTTCGACTCGTCCGTAG | TGGACCAGAAAGTGATGCGT |
| AT5G40010 | ChIP-q-PCR | CAAATTGGTCAGCACACATG | GAAGAAGGTAGTGTGTATAC |
